# Revision of “*Balaena*” *belgica* reveals a new right whale species, the possible ancestry of the northern right whale, *Eubalaena glacialis*, and the ages of divergence for the living right whale species

**DOI:** 10.7717/peerj.3464

**Published:** 2017-06-27

**Authors:** Michelangelo Bisconti, Olivier Lambert, Mark Bosselaers

**Affiliations:** 1 San Diego Natural History Museum, San Diego, CA, USA; 2 Royal Belgian Institute of Natural Sciences, Brussels, Belgium; 3 Zeeland Royal Society of Sciences, Middelburg, the Netherlands

**Keywords:** Cetacea, Balaenidae, *Eubalaena ianitrix*, Mysticeti, Phylogeny, Pliocene

## Abstract

In 1941, Abel established *Balaena belgica* based on a series of fused cervical vertebrae and citing other cranial fragments from the late Neogene of the Antwerp harbor (northern Belgium). Later, Plisnier-Ladame & Quinet (1969) added a neurocranium and other skeletal remains from the same area to this species. Recently, the neurocranium was re-assigned to the genus *Eubalaena* thanks to newer phylogenetic analyses. Here, a new description is provided of materials previously assigned to “*Balaena*” *belgica* together with taxonomic revisions. Our work suggests that the cervical complex originally designated as the type of “*Balaena*” *belgica* is too poorly preserved to be used as such and is assigned to Balaenidae gen. et sp. indet., thus making “*Balaena*” *belgica* a nomen dubium. In addition to the neurocranium, the other remains consist in a fragment of maxilla assigned to Balaenidae gen. et sp. indet. and in a humerus assigned to *Eubalaena* sp. Discovered in the Kruisschans Sands Member of the Lillo Formation (3.2–2.8 Ma, Piacenzian, Late Pliocene), the neurocranium is designated as the holotype of the new species *Eubalaena ianitrix*. Our phylogenetic analysis supports a sister-group relationship of *Eubalaena ianitrix* and *Eubalaena glacialis*, and helps constraining the ages of origin for balaenid clades. Ecological and phylogenetic data suggest that *Eubalaena ianitrix* may represent the direct ancestor of *Eubalaena glacialis*, the latter having evolved through phyletic transformation including body size increase during the temperature decline of the Late Pliocene.

## Introduction

Living right whales include North Atlantic, southern and North Pacific right whales, all of them grouped within the genus *Eubalaena* (Cetacea, Mysticeti, Balaenidae; Kenney, 2009; Rice, 2009). The North Atlantic or northern right whale obviously inhabits the North Atlantic Ocean, the southern right whale is distributed in the waters around Antarctica, and the North Pacific right whale is present in a portion of the Pacific that is limited in the south by southern Japan and the southern portion of the California peninsula (Kenney, 2009). Recent studies have addressed molecular taxonomy, population dynamics, and distribution patterns of these whales suggesting that the genus *Eubalaena* should include three species corresponding to the three groups mentioned above (namely, *Eubalaena glacialis*, *Eubalaena australis* and *Eubalaena japonica*) (Rosenbaum et al., 2000). Although a full agreement on this point has not been reached yet, it is largely acknowledged that North Atlantic and North Pacific right whales are suffering high extinction risk (Clapham, Young & Brownell, 1999). This is probably due to the catastrophic bottleneck effect induced into their populations by human hunting activities during 19th and 20th centuries (Gaskin, 1986) that drastically reduced the size of their populations in a brief period.

The assessment of the genetic diversity of the living right whale populations largely depends on the reconstruction of the population size before the start of industrial whaling (Rooney, Honeycutt & Derr, 2001; Rosenbaum et al., 2000; Malik et al., 2000). Such a reconstruction depends on several factors including the phylogenetic history of the genus and divergence time from the living species that is phylogenetically closest to the living right whales (Rooney, Honeycutt & Derr, 2001), namely the bowhead whale *Balaena mysticetus*. The study of the fossil record may help determining the antiquity of the genus *Eubalaena* and constraining the time of divergence of *Eubalaena* from the bowhead whale (McLeod, Whitmore & Barnes, 1993; Santangelo et al., 2005).

The fossil record of *Eubalaena* is scanty and scattered around the northern hemisphere. A right whale skull from the Pleistocene of Japan was described by Nishiwaki & Hasegawa (1969) and reviewed by Kimura (2009). Kimura (2009) also described *Eubalaena shinshuensis* from the latest Miocene of the Gonda Formation, Nagano Prefecture, Japan. A partial skull of an indeterminate species of *Eubalaena* was described by Bisconti (2002) from the Upper Pliocene of Tuscany, Central Italy. Fragmentary tympanic bullae assigned to *Eubalaena* spp. were described by Morgan (1994) from the Nashua Formation in Florida (latest Pliocene and earliest Pleistocene) and Boessenecker (2013) from the Purisima Formation in Central California (Late Pliocene). Finally, Field et al. (2017) described a fragmentary skull assigned to *Eubalaena* sp. from the Tjorres Formation in Island (Early Pliocene).

A large-sized balaenid skull from the “Merxemien” of Antwerp, northern Belgium, was described by Plisnier-Ladame & Quinet (1969) who assigned it to *Balaena belgica*, a taxon established by Abel (1941) based on a described and illustrated cervical complex and the mention of other cranial remains. Bisconti (2003) questioned Abel’s taxonomic decision and suggested that the skull should be assigned to *Eubalaena*, a proposal supported by later phylogenetic analyses placing “*B.” belgica* as sister-group to *Eubalaena glacialis* (Bisconti, 2005a; Churchill, Berta & Deméré, 2012) or as sister-group to the extant *Eubalaena* species (Marx & Fordyce, 2015). However, a formal re-description of the specimen is currently necessary to make sound taxonomic decisions.

**Figure 1:**
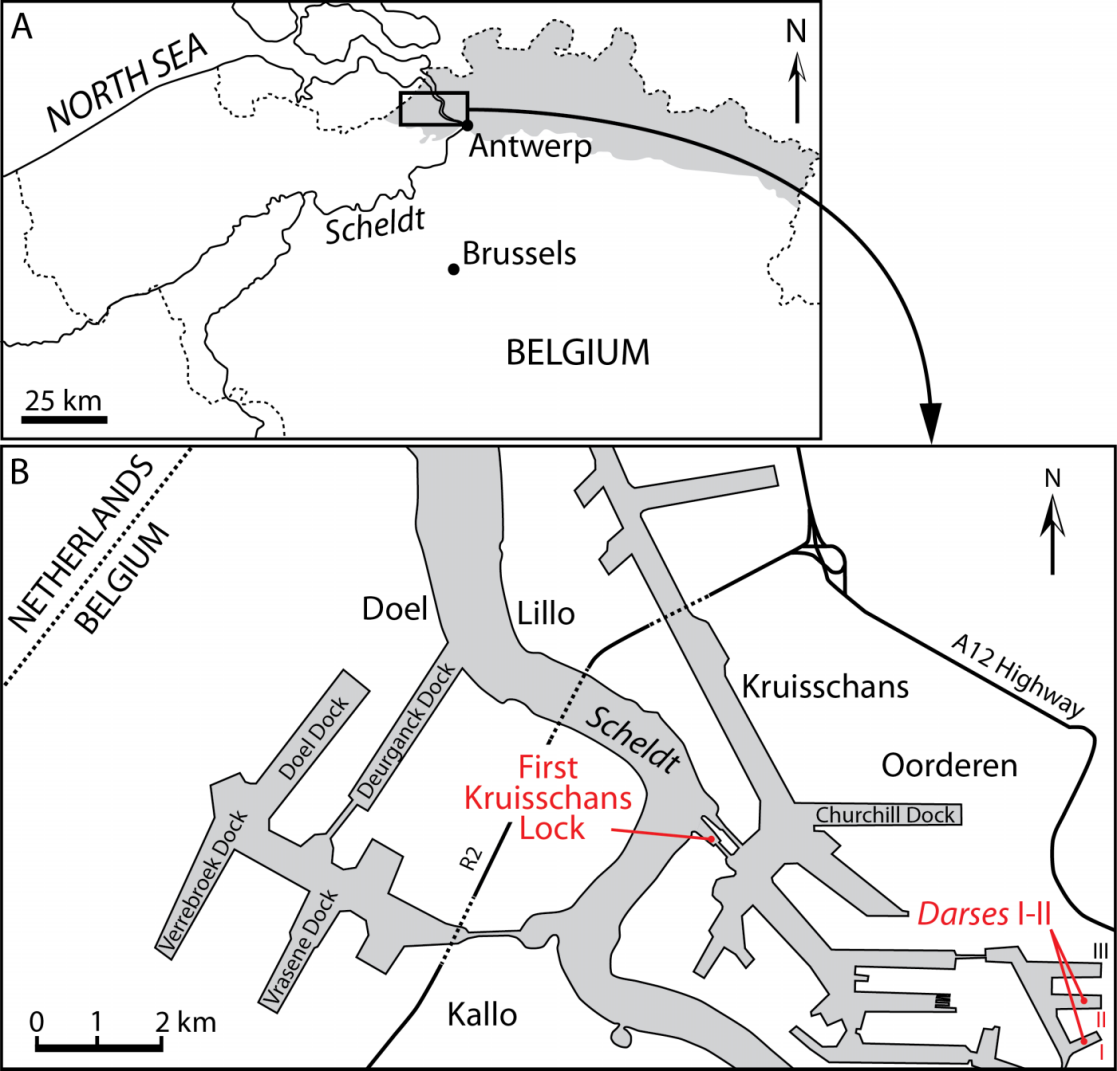
Localities of the balaenids described in this paper. (A) Localization of Antwerp in Belgium and its relationships with the North Sea. Gray whading represents marine Pliocene deposits. (B) Detailed map of the Antwerp harbor showing the first Kruisschans lock, where the holotype of *Eubalaena ianitrix* sp. nov. (RBINS M. 879a-f) and the fragment of maxilla RBINS M. 880 were found. The cervical vertebrae RBINS M. 881 were discovered in the “Darses I–II” in Oorderen. Modified from De Schepper, Head & Louwye (2009).

**Figure 2:**
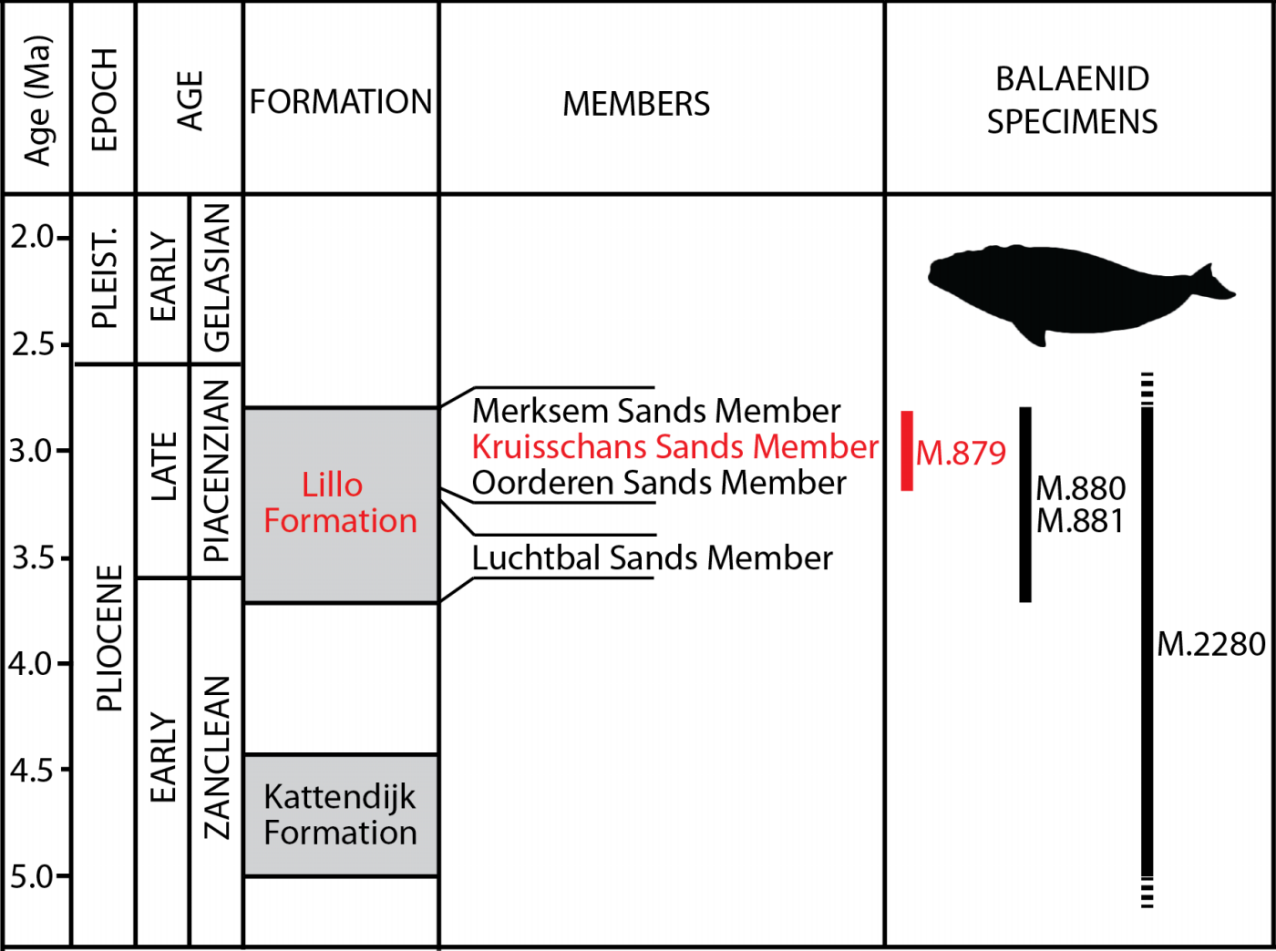
Lithological units from the Pliocene of the Antwerp area. Formations, members and their ages are provided, including the Kruisschans Sands Member of the Lillo Formation in the Piacenzian (Late Pliocene), where the holotype of *Eubalaena ianitrix* sp. nov. RBINS M. 879a-f was discovered. Modified from De Schepper, Head & Louwye (2009).

The specimens previously assigned to “*Balaena*” *belgica* consist of:

1. A cervical vertebrae complex discovered on March 6th 1914 by G. Hasse in the docks of the Antwerp harbor, figured by Abel (1941, pl. 2, fig. 9) and Plisnier-Ladame & Quinet (1969, fig. 1, pls. 1 and 2), and bearings the inventory number of the Royal Belgian Institute of Natural Sciences, Brussels (hereinafter RBINS) RBINS M. 881 (IG 8444);
2. A partial neurocranium (RBINS M. 879a-f, IG 8652) discovered in 1921 in Oorderen (a part of the Antwerp harbor) during the excavation of the first Kruisschans lock (Figs. 1 and 2), figured by Plisnier-Ladame & Quinet (1969, pls. 1–2);
3. A large fragment of right maxilla (RBINS M. 880a-c, IG 8652) also discovered in 1921 in Oorderen during the excavation of the first Kruisschans lock seemingly misidentified as a fragment of mandible by Plisnier-Ladame & Quinet (1969), but never described or figured;
4. A large isolated left humerus (RBINS M. 2280) without any locality data, most likely corresponding to the specimen mentioned by Plisnier-Ladame & Quinet (1969), but never described or figured.

In this paper, the material previously assigned to “*Balaena*” *belgica* by Abel (1941) is newly described and compared with an extended sample of right, bowhead and pygmy right whales to get a comprehensive analysis of anatomy and clear taxonomic assignments. The morphological characters of the skull are then used in a new phylogenetic analysis of living and fossil right and bowhead whales to (1) reveal the timing of the origin of the genus *Eubalaena* and the divergence time from its closest living relative *Balaena mysticetus* and (2) investigate whether the three living right whale populations correspond to three different species confirming or not the results of molecular analyses. Our results will hopefully provide molecular ecologists with useful information for safer reconstructions of past population dynamics of these highly endangered species.

## Materials and Methods

### New species name

The electronic version of this article in portable document format (PDF) will represent a published work according to the International Commission on Zoological Nomenclature (ICZN), and hence the new names contained in the electronic version are effectively published under that code from the electronic edition alone. This published work and the nomenclatural acts it contains have been registered in ZooBank, the online registration system for the ICZN. The ZooBank LSIDs (life science identifiers) can be resolved and the associated information viewed through any standard web browser by appending the LSID to the prefix http://zoobank.org/. The LSID for this publication is: urn:lsid:zoobank.org:pub:C8D3FE95-303E-4EF4-86DD-1B453E124981. The online version of this work is archived and available from the following digital repositories: PeerJ, PubMed Central1 and CLOCKSS.

### Anatomy

Anatomical terms for skull osteology follow Mead & Fordyce (2009); terminology for humerus and cervical vertebrae follows Schaller (1999).

### Comparative analyses

Comparative analyses were made with an extended balaenoid sample including specimens from museums MSNT, RBINS, AMNH, NBC and IZIKO (specimens are listed in Bisconti (2011)). In addition, specimens described in literature were used to complement first-hand observations (True, 1904; Omura, 1958; Tomilin, 1967; Tsai & Fordyce, 2015).

### Body size estimate

Three methods for body size estimate were followed. First, we used the regression equation provided by Pyenson & Sponberg (2011) that allows the reconstruction of the total body length based on a measure of the bizygomatic width of the skull. The equation is the following (data in mm):

(1) $$\log ({\rm{total}}\;{\rm{body}}\;{\rm{length}}) = 0.92({\rm{log}}({\rm{bizygomatic\;width}}) - 1.64) + 2.67$$

Pyenson & Sponberg (2011) used this equation to reconstruct total body lengths of living and fossil cetaceans including mysticetes. Unfortunately, their study did not involve balaenid specimens, therefore we cannot be sure that the Eq. (1) is well suited to provide an accurate reconstruction of the total body length for Balaenidae. Moreover, results from Eq. (1) deviated from observed values of intact specimens for amounts ranging from 47% to 37%. Bearing this in mind, we corrected results generated by the Eq. (1) by reducing our results by 47% and 37%; in so doing, we got two results from Eq. (1) corresponding to the range of estimates for the total body length of RBINS M. 879a-f.

The second method used the occipital breadth as principal predictor as from the following equation, provided by Evans et al. (2012) (measurements in mm):

(2) $${\rm{Body\;mass}} = 4.924*{10^{ - 6}}{\left( {{\rm{occipital\;breadth}}} \right)^{3.858}}$$

The Eq. (2) showed a high correlation coefficient in mammals (*R*^2^ = 0.9447). Once a body mass estimate was obtained, we used Eq. (3) to obtain an estimate of skeletal length. Eq. (3) is the following, as developed by Silva & Downing (1995):

(3) $$\log \left( {{\rm{body\;mass}}} \right) = 3.08\left( {\log \left( {{\rm{skeletal\;length}}} \right)} \right) - 4.84$$

This equation was extensively used in the reconstructions of body masses and skeletal lengths of living and fossil mammals in previously published papers. Unfortunately, in marine mammals, body mass may change during the life cycle depending on different patterns of activity performed in the year (e.g., foraging, migration, female lactation, etc.) thus the body mass estimate provided by Eq. (3) is to be intended as mean body mass for a whale of a given length (Churchill, Clementz & Kohno, 2014).

Unfortunately, none of these equations was tested on balaenid records and it is not known if they are actually able to retrieve correct results in this family. For this reason, we used also the regression equation provided by Bisconti (2002) to predict the total skull length of a balaenid whale based on supraoccipital length. The equation is the following:

(4) $${\rm{Supraoccipital\;length}} = 0.3937\left( {{\rm{skull\;length}}} \right) - 62.803$$

In this equation, skull length corresponds to condylobasal length. Unfortunately, the correlation coefficient associated to this equation is rather low (*R*^2^ = 0.5967) because the regression equation is based on a limited and scattered dataset. Once a condylobasal length is obtained, we inferred the total body length by tripling or quadrupling the condylobasal length. In fact, following Tomilin (1967), the skull length is about 25-to-30% of the total body length in extant Balaenidae. Presently it is not possible to be sure that this proportion applies to fossil balaenids; however, given that skull and body sizes have important adaptive functions in Balaenidae (Sanderson & Wassersug, 1993), and given that RBINS 879a-f represents an advanced balaenid species (as judged from its placement in the phylogenetic hypothesis of relationships presented in this paper), there is no reason to propose a fundamentally different skull/body ratio in this specimen.

### Phylogenetic analysis

A total of 153 morphological characters were coded for 42 taxa including three archaeocetes used as outgroups. The taxonomic sampling adopted here includes representative taxa from all the known mysticete radiations. The family Balaenidae was represented by 11 taxa including *Morenocetus parvus*; Neobalaenidae was represented by *Caperea marginata* and *Miocaperea pulchra*. The Pliocene *Eubalaena* sp. from Tuscany was included in a phylogenetic analysis for the first time.

Characters were coded based on direct examination of specimens and on the literature listed in the Supplementary Information together with both character list and taxon x character matrix. Only two characters were coded from baleen morphology; all the other characters were coded from the analysis of the skeletal anatomy of mysticetes and archaeocetes. All characters were unordered and unweighted and followed the outgroup polarization criterion.

Character choice was made bearing in mind the goal of maximum reduction of homoplasy in the dataset. This goal was achieved by examining the homoplasy level shown by each character states published by Bisconti (2008, 2011), Bisconti, Lambert & Bosselaers (2013), Bisconti & Bosselaers (2016), Marx (2011) and Boessenecker & Fordyce (2015). Bisconti, Lambert & Bosselaers (2013) and Bisconti & Bosselaers (2016) published the consistency index (hereinafter abbreviated as CI) of all the synapomorphies supporting named nodes. Characters with CI < 1 were considered homoplastic and were excluded from the present dataset. As far as characters from other papers are concerned, it was more difficult to decide whether a character had a homoplastic distribution or not. To get decisions, character states were mapped on published phylogenetic hypotheses and their distributions were assessed by eye; in the case a character showed scattered distribution across the branches of the Mysticeti tree, then the application of Fitch’s (1971) parsimony allowed to decide if the character could be considered homologous or not in those branches.

The taxon x character matrix was treated by TNT (Goloboff, Farris & Nixon, 2008) with default parameters for new technology search. The synapomorphies were mapped onto the resulting cladogram and were listed through the dedicate commands in TNT. Number of steps added by each character was calculated at relevant nodes to determine whether the character state constituted an ambiguous or unambiguous synapomorphy at the node.

### Stratigraphic consistency index and determination of divergence dates

The degree of agreement between the branching pattern and the stratigraphic occurrence of the taxa was assessed by the calculation of the stratigraphic consistency index (hereinafter, SCI) following the method described by Huelsenbeck (1994; see also discussion in Bisconti (2007)). Stratigraphic ages of the taxa were obtained from the paleobiology database available at https://paleobiodb.org and mainly compiled by Mark D. Uhen. Adjustments to the ages of the specimens provided by Marx & Fordyce (2015) were also included where necessary. Stratigraphic ages of the taxa are provided in the Supplementary Information published in the website of this Journal. The stratigraphic intervals of occurrence of the taxa were used to constrain the divergence dates of the branches included within Balaenoidea in order to get information about the origin of the living right whale and bowhead whale species.

## Systematic Paleontology

Class MAMMALIA Linnaeus, 1758
Order CETACEA Brisson, 1762
Clade PELAGICETI Uhen, 2008
Clade NEOCETI Fordyce & de Muizon, 2001
Suborder MYSTICETI Cope, 1891
Infraorder CHAEOMYSTICETI Mitchell, 1989
Parvorder BALAENOMORPHA Geisler & Sanders, 2003
Superfamily BALAENOIDEA Flower, 1864
Family BALAENIDAE Gray, 1825
Balaenidae gen. et sp. indet.

**Material**: Cervical vertebrae complex RBINS M. 881 (IG 8444). This specimen was first figured and described as the cotype of *Balaena belgica* by Abel (1941, p. 13; pl. 2, fig. 9), and later commented and re-illustrated by Plisnier-Ladame & Quinet (1969, fig. 1; pl. 1 and 2, associated to neurocranium RBINS M. 879).

**Locality and horizon information:** The specimen was found on March 6, 1914 by G. Hasse in the docks of Antwerp harbor (northern Belgium), more precisely in the “darses I-II” (Fig. 1). Abel (1941) mentions an origin in the “Scaldisien” for this specimen. Now disused, this chronostratigraphic regional unit is roughly equivalent to the Lillo Formation, a latest early to Late Pliocene lithostratigraphic unit (latest Zanclean to Piacenzian; Laga, Louwye & Mostaert, 2006; De Schepper, Head & Louwye, 2009; see Fig. 2).

**Figure 3:**
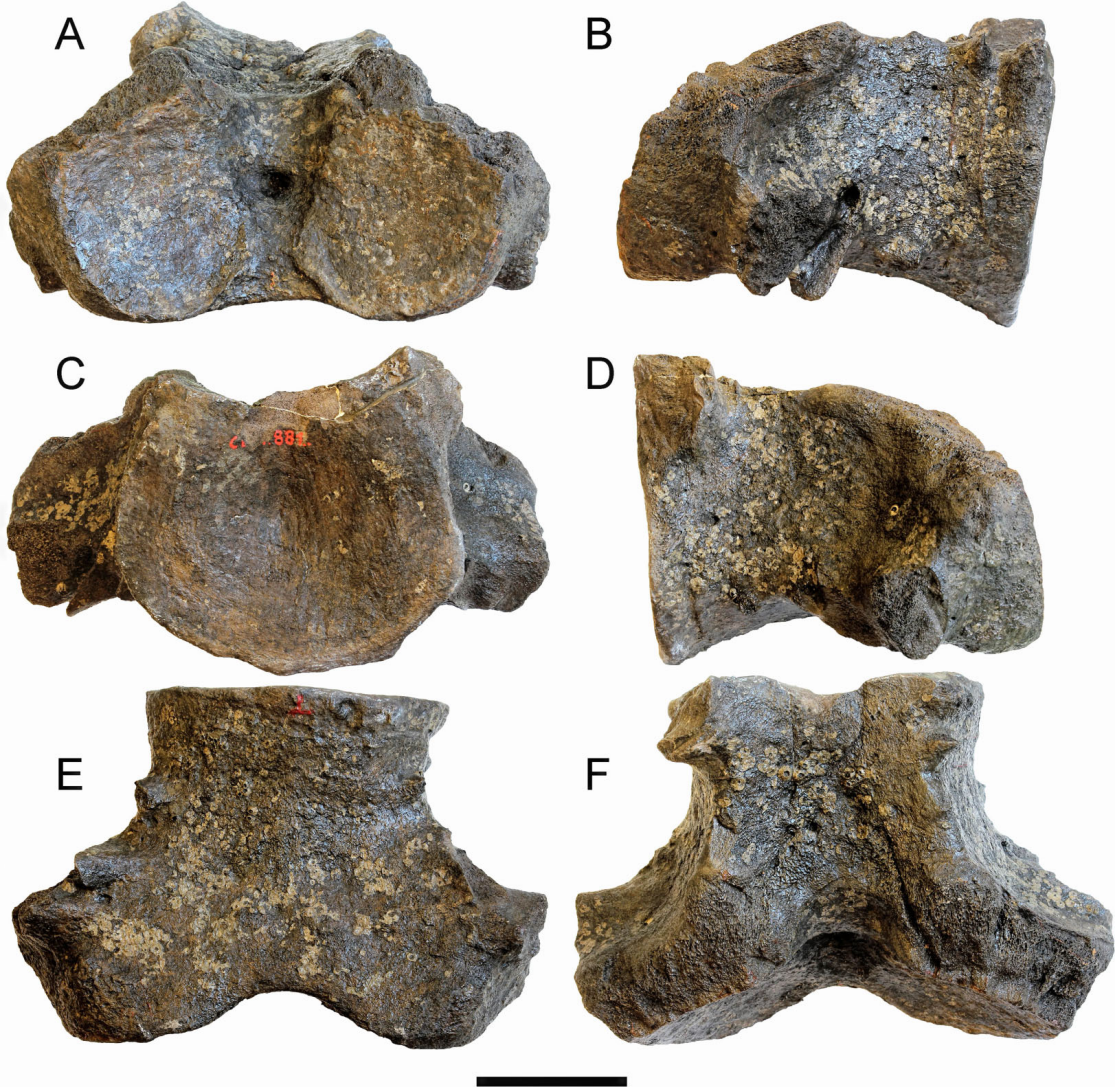
The cervical vertebrae RBINS M. 881 that were originally used as type of “*Balaena*” *belgica* by Abel (1941) and are reassigned to Balaenidae gen. et sp. indet. in this work. (A) anterior view, (B) left lateral view, (C) posterior view, (D) right lateral view, (E) ventral view, (F) dorsal view. Scale bar equals 10 cm.

**Description:** The specimen includes a complex formed by fused cervical vertebrae (Fig. 3). Anteriorly, only the ventral portions of the articular facets of the atlas for the occipital condyles of the skull are preserved. The articular surfaces of the facets are highly concave and wide (measurements are provided in Table 1). The articular facets are separated dorsally by a wide concavity that corresponds to the ventral border of the neural canal. Posteriorly, the articular facet of the seventh cervical vertebra for the first thoracic vertebra is highly concave and shows a uniformly convex lateral border. Laterally, the ventral apophysis of the atlas protrudes laterally and ventrally and is separated from a small fragment of the ventral apophysis of the axis by a narrow, dorsoventral groove that is slightly oblique in lateral view. The transverse grooves that are sometimes observed in the cervical complexes of *Caperea marginata* and in balaenid species (Bisconti, 2012) are not seen in this specimen. No additional characters can be described due to the poor preservation of the specimen.

**Table 1:** Measurements (in mm) of RBINS M. 880 (cervical vertebrae complex, Balaenidae gen. et sp. indet.) and M. 2280 (left humerus, *Eubalaena* sp.).

Character	Measure
M. 880 (cervical vertebrae)	
Maximum anteroposterior length of whole complex	280
Maximum transverse width of whole complex	423
Maximum width across articular facets of atlas	384
Maximum height of articular facets of atlas	175
Posterior width of centrum of last cervical	246
Posterior height of centrum of last cervical	201
M. 2280 (left humerus)	
Total length	683
Maximum proximal mediolateral width	355
Maximum proximal anteroposterior width	458
Anteroposterior diameter of humeral head	345
Mediolateral diameter of humeral head	343
Minimum mediolateral width of diaphysis	222
Minimum anteroposterior width of diaphysis	271
Distal mediolateral width	249
Maximum distal anteroposterior width	364
Anteroposterior length of radial facet	239
Anteroposterior length of ulnar facet (including facet for olecranon)	250

**Note:**

Characters are measured as preserved.

Moran et al. (2014) published a study on the ontogenetic fusion of the cervical vertebrae in the extant bowhead whale *Balaena mysticetus*, observing that total fusion of the vertebral centra in the cervical region occurs between 10 and 20 years after birth. In RBINS M. 881 the fusion appears complete as the grooves observed at the dorsolateral and ventrolateral corners of the cervical complex are not deep and do not allow to separate the centra. It is thus possible that RBINS M. 881 belonged to an individual of an age included between 10 and 20 years. However, this hypothesis should be tested with comparisons to the fusion pattern of vertebral centra in the cervical region of *Eubalaena* in a way to get a more accurate estimate of the individual age of this specimen. Unfortunately, such a study is still lacking.

**Discussion and taxonomic decision:** The specimen represents a complex that presumably includes all the cervical vertebrae of a balaenid whale. The morphology is consistent with that of Balaenidae as in *Caperea marginata* the ventral apophysis projects much more ventrally and the outline of the posterior articular surface of the seventh cervical vertebra is squared in posterior view. In other mysticetes the cervical vertebrae are not fused; fusion may occasionally occur in the presence of pathological processes, but the involvement of all the cervical vertebrae is extremely rare. It is possible to distinguish the cervical vertebrae of the living species of *Eubalaena* from the extant *Balaena mysticetus* based on: (1) shape of the neural apophysis, (2) shape of the neural canal and (3) size, shape and orientation of the ventral apophysis of the atlas. Unfortunately, the specimen RBINS M. 881 is too poorly preserved to allow a safe identification; in fact, in this specimen the neural apophyses are not preserved, the neural canal is only partly preserved, and the ventral apophyses of the atlas are largely damaged and worn. For this reason, we assign RBINS M.881 to Balaenidae gen. et sp. indet. Consequently, this decision implies that this specimen cannot be designated as the holotype of the species *Eubalaena belgica*. Therefore, as Abel (1941) designated RBINS M. 881 as the cotype of “*Balaena*” *belgica* and now we assign it to gen. et sp. indet., it follows that both *“Balaena” belgica* and its recombination, *Eubalaena belgica*, are nomina dubia.

Balaenidae gen. et sp. indet.

**Material:** Fragment of right maxilla RBINS M. 880a-c (IG 8652), mentioned as a fragment of mandible by Plisnier-Ladame & Quinet (1969, p. 2), but never figured.

**Locality and horizon information:** The specimen was found at Oorderen during the excavation of the first Kruisschans lock of the Antwerp harbor at a depth of 7.80 m under the sea level (Fig. 1). The specimen originates from the Lillo Formation (“Scaldisien”), in a level slightly lower than the neurocranium RBINS M. 879 (see below). Its geological age falls in the range 3.7–2.8 Ma (latest Zanclean-Piacenzian; De Schepper, Head & Louwye, 2009; Fig. 2).

**Figure 4:**
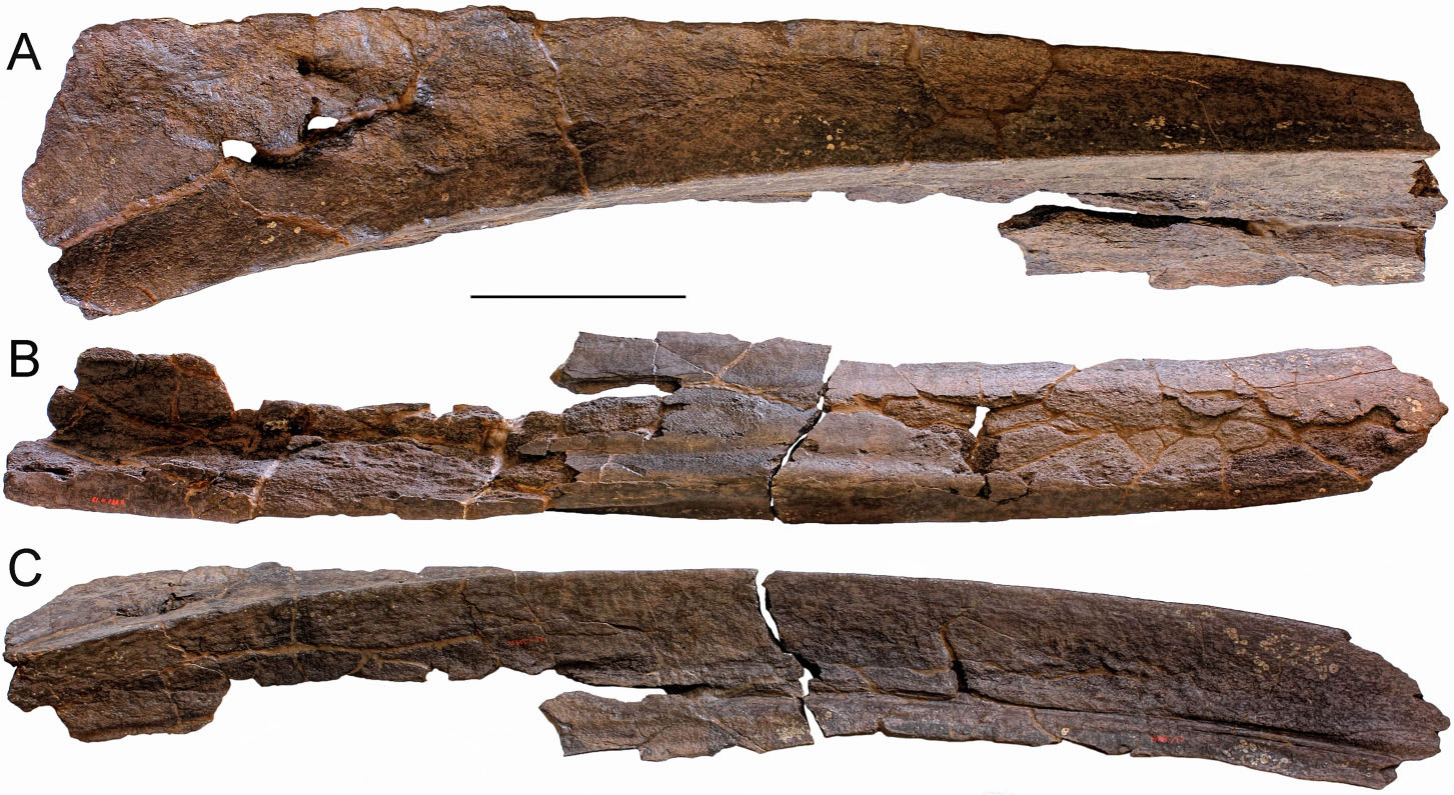
The fragment of right maxilla RBINS M. 880 assigned to Balaenidae gen. et sp. indet. in this work. (A) Dorsolateral view, (B) dorsomedial view, (C) ventromedial view. Scale bar equals 30 cm.

**Description:** The specimen includes part of the proximal portion of the right maxilla of a balaenid whale (measurements are provided in Table 1). The maxilla is transversely compressed and bears an arched and thin lateral border (Fig. 4). Posteriorly, three infraorbital foramina are observed; ventrally a long groove for the vasculature of the baleen-bearing tissue runs along the whole ventral surface of the bone. Such a surface is lateromedially and anteroposteriorly concave. It is not clear if the orientation of this fragment is more similar to *Eubalaena* and *Balaenula* (in these taxa the posterior portion of the maxilla is nearly horizontal in lateral view) or to *Balaena mysticetus* (in this species the posterior portion of the maxilla projects dorsally and anteriorly) or to *Balaenella brachyrhynus* (in this species the posterior portion of the maxilla distinctly projects anteroventrally).

**Discussion and taxonomic decision**: The specimen RBINS M. 880a-c represents a balaenid maxilla. In fact it shows a distinctive arch in lateral view, it is transversely compressed, and it displays a longitudinally developed groove for the vasculature of the baleen-bearing tissue. Unfortunately, it is impossible to reconstruct the original orientation of this fragment in the skull; this, together with the lack of the anterior portion of the rostrum and of the lateral process of the maxilla, prevents a safe taxonomic assignment. For this reason, we assign RBINS M. 880a-c to Balaenidae gen. et sp. indet.

Genus *Eubalaena* Gray, 1864
*Type species. Eubalaena australis* Desmoulins, 1822.

**Holotype:** An unnumbered skeleton housed at the Museum National d’Histoire Naturelle, Paris, France.

**Diagnosis of genus**: Balaenid cetacean characterized by all the characters diagnostic of the *Eubalaena + Balaenula* clade (i.e., rostrum and supraorbital process of the frontal form a right angle in lateral view, nasal and proximal rostrum horizontal in lateral view, orbitotemporal crest well developed on the dorsal surface of the supraorbital process of the frontal, and zygomatic process of the squamosal directed anteriorly so that the posterior wall of the temporal fossa cannot be observed in lateral view) and by the following, exclusively *Eubalaena* characters: vertically oriented squamosal, protruding lambdoid and temporal crests, convex and protruding supramastoid crest, dome-bearing supraoccipital, wide and rounded anterior process of supraoccipital, and pars cochlearis of petrosal protruded cranially.

**Discussion:** Bisconti (2003) provided the last diagnosis of *Eubalaena* published up to the present work; diagnostic characters included: gigantic body size (maximum body length approaching 22 m), rostrum and supraorbital process of frontal form a right angle, nasal and proximal rostrum horizontal, ascending temporal crest well developed on the dorsal surface of the supraorbital process of the frontal, vertically developed squamosal, zygomatic process of the squamosal directed anteriorly so that the posterior wall of the temporal fossa cannot be observed in lateral view, protruding lambdoidal and temporal crests, convex and protruding lateral squamosal crest, exoccipital squared in lateral view, dome-bearing supraoccipital shield with sagittal crests, wide anterior process of supraoccipital, pars cochlearis cranially protruding, and superior process of petrosal cranially protruding. Bisconti’s (2003) diagnosis is certainly useful to separate extant *Eubalaena* from other living balaenids but it may be of limited help when trying to separate fossil *Eubalaena* species from other living and fossil balaenids. In particular, the above diagnosis includes characters that are shared with the extinct *Balaenula* lineage: rostrum and supraorbital process form a right angle, nasal and proximal rostrum horizontal, ascending temporal crest (orbitotemporal crest *sensu* Mead & Fordyce, 2009) well developed on the dorsal surface of the supraorbital process of the frontal, and exoccipital squared in lateral view. All these characters can be observed also in *Balaenula astensis* or in *Balaenula balaenopsis*. A more detailed diagnosis of *Eubalaena* allowing to separate this genus from all the other living and extinct balaenid taxa includes the characters listed in the Emended diagnosis of genus provided above.

*Eubalaena* sp. indet.

**Material:** Left humerus RBINS M. 2280, mentioned by Plisnier-Ladame & Quinet (1969, p. 2), but never figured.

**Locality and horizon information:** Antwerp area. There is no precise locality data available for this specimen. A stratigraphic assessment is currently impossible.

**Figure 5:**
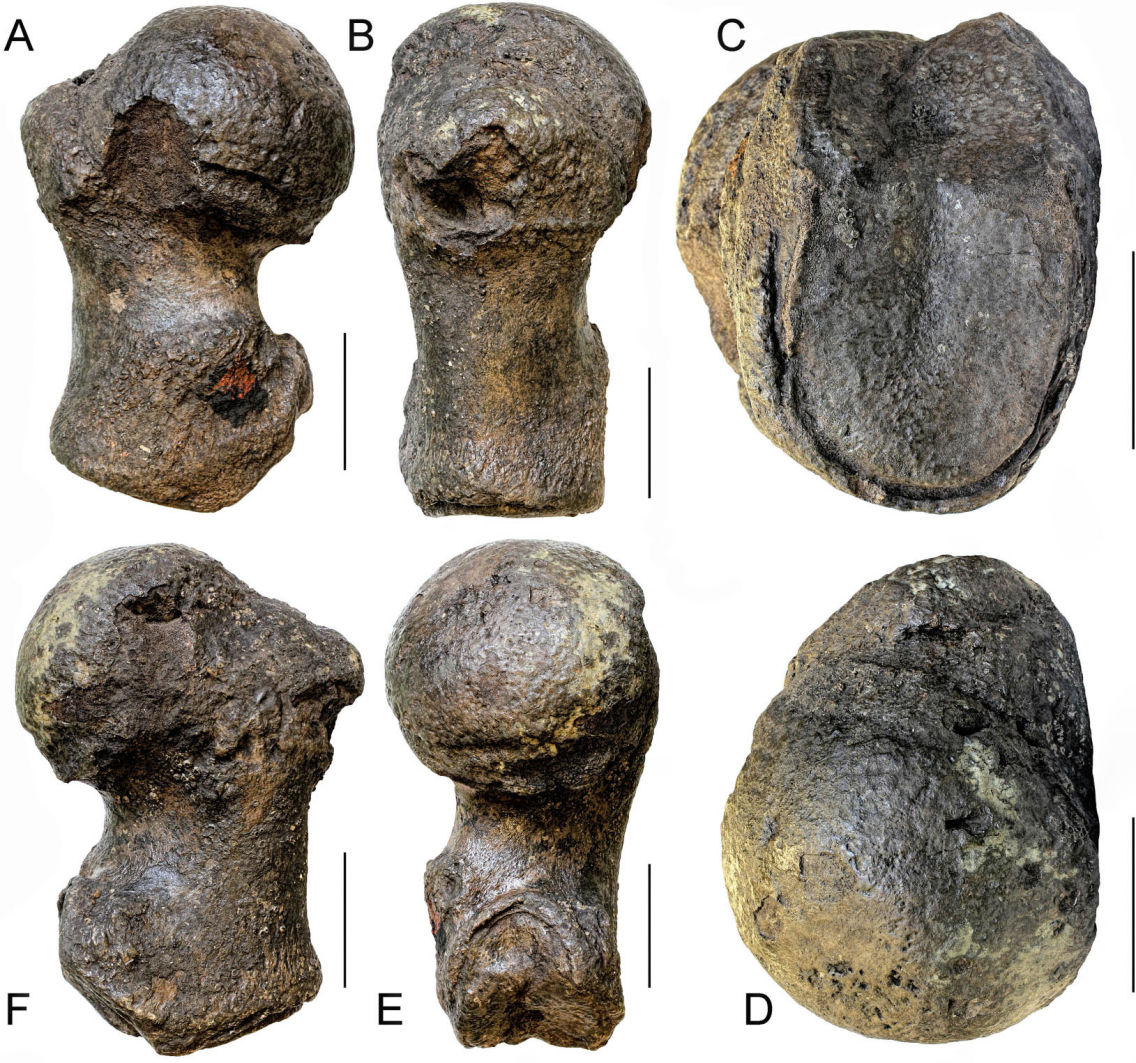
The left humerus RBINS M. 2280 assigned to *Eubalaena* sp. in this work. (A) Lateral view, (B) anterior view, (C) distal view of articular facets for radius and ulna, (D) proximal view or articular head for scapula, (E) posterior view, (F) medial view. Scale bars equal 10 cm.

**Description:** This well-preserved, robust left humerus shows a highly rounded proximal articular head that is anteriorly bounded by a protruding deltoid tuberosity; the latter is triangular in lateral view (measurements are provided in Table 1). The diaphysis shows straight anterior and posterior borders (Fig. 5); the posterior border is shorter than the anterior border, as it terminates more proximally due to the development of the articular facet for the olecranon process of the ulna. Such a facet protrudes posteriorly and occupies part of the posterior border of the humerus. The anteroventral corner of the humerus protrudes anteriorly forming a kind of triangular tuberculum. The articular facets for radius and ulna are separated by a transverse protrusion that is triangular in lateral view.

**Discussion and taxonomic decision:** The morphology of the articular head of the humerus RBINS M. 2280 is consistent with both *Eubalaena* and *Balaena*. In *Eubalaena glacialis* the external border of the lateral surface of the articular head shows a posterior concavity that is not seen in *Balaena mysticetus* (Benke, 1993). Unfortunately, RBINS M. 2280 is worn in that region thus preventing a clear understanding of its morphology. More distally, the articular facet for the olecranon is well developed as seen in the extant *Eubalaena* species while in *Balaena mysticetus* it is largely reduced. Benke (1993) showed that the posterior border of the diaphysis in *Balaena mysticetus* is uniformly concave and short and that the deltoid tuberosity is less protruding than in *Eubalaena glacialis*. In the latter, the posterior border of the diaphysis is more elongated (resembling that of RBINS M. 2280) and the deltoid tuberosity is triangular and protruding. In the humerus RBINS M.2280 the deltoid tuberosity is triangular and protruding as in *Eubalaena glacialis*. However, the posterior border of the diaphysis of RBINS M. 2280 is straighter than that observed in *Eubalaena glacialis*.

Comparative analysis shows, thus, that the humerus RBINS M. 2280 is closer to *Eubalaena* than to *Balaena*, as it shares with *Eubalaena glacialis* the presence of (1) well developed and protruding articular facet for the olecranon process, (2) triangular and protruding deltoid tuberosity and (3) comparatively long posterior border of the diaphysis. These shared characters allow inclusion of RBINS M. 2280 within *Eubalaena*. However, the different shape of the posterior border of the diaphysis and the lack of information about the shape of the lateral outline of the articular head do not allow inclusion of this specimen within *Eubalaena glacialis* or other extant *Eubalaena* species. RBINS M. 2280 is thus assigned to *Eubalaena* sp. indet.

When compared to the extant *Eubalaena* species, this humerus is particularly long suggesting that it belonged to a large individual. The total proximodistal length of RBINS M. 2280 is 683 mm, which is greater than the maximum humeral lengths published by Benke (1993) for *Balaena mysticetus* (605 mm), *Eubalaena glacialis* (555 mm) and *Eubalaena australis* (619 mm), and by Omura (1958) for *Eubalaena japonica* (556 mm). Based on this comparison, we suggest that the humerus RBINS M. 2280 belonged to an individual that was longer than 16.5 m. This is the first report of a gigantic right whale in the fossil record of the North Sea.

*Eubalaena ianitrix* sp. nov. LSID: urn:lsid:zoobank.org:act:F17C4DCA-FF1B-4EA4-9E6B-6C1EED448745

**Derivation of name:** The specific name *ianitrix* derives from Ianus, the Roman God who was the guardian of passages, gates and doors. This name is related to the discovery of the holotype in the locks (or entrances) of the Antwerp harbor.

**Holotype:** The holotype is housed at the Royal Belgian Institute of Natural Sciences, Brussels, Belgium, and bears the inventory number M. 879a-f, Reg. 4019, I.G. 8652 (all the numbers refer to the same individual). It includes a partial skull (M. 879a), right squamosal and exoccipital (M. 879b), left squamosal and exoccipital (M. 879c), fragment of a maxilla (M. 879d), fragment of the right supraorbital process of the frontal (M. 879e), fragment of the left supraorbital process of the frontal (M. 879f). It was first figured and described as *Balaena belgica* by Plisnier-Ladame & Quinet (1969, p. 2; pl. 1–2, associated to cervical complex RBINS M. 8811).

**Type locality:** The neurocranium RBINS M. 879a-f was discovered in Oorderen (Fig. 1) during the excavation of the first Kruisschans lock (“*première écluse du Kruisschans*,” now named Van Cauwelaertsluis) of the Antwerp harbor (Plisnier-Ladame & Quinet, 1969). Geographic coordinates: 51°16′32″N–04°19′51″E. As mentioned above, the maxilla RBINS M. 880a-c was found at the same site. However, based on labels associated to specimens, the neurocranium was found at a depth of 3.70 m under the sea level, whereas the maxilla was found at a depth of 7.80 m under the sea level, therefore most likely not representing the same individual.

**Type horizon:** Based on data associated to the neurocranium RBINS M. 879a-f, Misonne (1958) indicated an origin in the Kruisschans Sands (“*Sables du Kruisschans*;” Fig. 2) in the “*zone à Cardium*,” and a Merksemian (“Merxemien”) stage, a stage assignation later confirmed by Plisnier-Ladame & Quinet (1969). Now disused, this regional stage was first introduced by Heinzelin (1955a), including the Kruisschans Sands and Merksem Sands, together with an underlying gravel layer (Laga, Louwye & Mostaert, 2006). Both the Kruisschans Sands Member and Merksem Sands Member are now part of the Lillo Formation, constituting its two youngest members (Vandenberghe et al., 1998; Laga, Louwye & Mostaert, 2006).

In published sections of the Pliocene and Quaternary layers at the Kruisschans locks (including sections in a new lock parallel to the ancient lock, “Ecluse Baudouin”), a clayey sand layer containing a high concentration of shells of the bivalve *Laevicardium* (first named *Cardium*) *parkinsoni* and isolated cetacean bone fragments is reported at a depth of 5.5–7 m (Heinzelin, 1952, 1955b). This shell layer is located about 1 m above the base of the Kruisschans Sands. It is therefore tempting to propose that the *“zone à Cardium”* mentioned by Misonne (1958) for the horizon of the skull RBINS M. 879a-f corresponds to this part of the Kruisschans Sands.

Dinoflagellate cysts from a section 4 km north to the Kruisschans locks give a Piacenzian (Late Pliocene) age to both the Kruisschans Sands Member and the overlying Merksem Sands Member, older than 2.6 Ma (as confirmed by pollens) and most likely somewhat younger than 3.7 Ma (age of the base of the Lillo Formation), whereas sequence stratigraphy narrows even more their temporal range to 3.2–2.8 Ma (De Schepper, Head & Louwye, 2009). RBINS M. 879a-f is therefore proposed to date from that Piacenzian interval.

The record of fossil marine mammals in the Kruisschans Sands Member is relatively poor; only the odobenid *Alachtherium antwerpiensis* and the stem phocoenid *Septemtriocetus bosselaersi* are known to originate from that unit (Hasse, 1909; Lambert, 2008).

**Diagnosis:** *Eubalaena ianitrix* differs from *Eubalaena shinshuensis* in showing a distinctive anteroventral corner in the parietal–frontal suture and in having an anterodorsally protruded squamosal–parietal suture; it differs from the *Eubalaena* sp. from the early Late Pliocene of Tuscany (included in our diagnosis considering that in our phylogenetic analysis it represents a true right whale species needing a new species name) in having an anteriodorsally protruded squamosal–parietal suture; it differs from *Eubalaena japonica* in having the pterygoid exposed in the temporal fossa, in having posteromedially directed anterior borders of the palatine and in having anteriorly directed posterior borders of the palatine; it differs from *Eubalaena australis* in having a less protruding anteroventral corner in the parietal–frontal suture, in having an anterodorsally protruded squamosal–parietal suture, in having the pterygoid exposed in the temporal fossa and in having anteriorly directed posterior border of the palatine; it differs from *Eubalaena glacialis* in having a crest located at the squamosal–parietal–supraoccipital suture and in having anteriorly directed posterior border of the palatine.

***Eubalaena ianitrix*:** Does not possess any autapomorphy and may be distinguished from other *Eubaena* species by the following combination of characters: bilateral bulge on supraoccipital with presence of sagittal crest, alisphenoid exposed in the temporal fossa, and alisphenoid dorsally bordered by a squamosal projection that prevents it to make contact with parietal.

### Comparative anatomy of the skull of *Eubalaena ianitrix*

The holotype specimen consists of a moderately well preserved partial skull. The skull is massive and heavy and lacks part of the supraoccipital borders due to post-mortem erosion. It is subdivided into six fragments that can be put together due to clear break surfaces. Measurements are provided in Table 2.

**Table 2:** Measurements (in mm) of the neurocranium RBINS M. 879a-f (holotype of *Eubalaena ianitrix* sp. nov.).

Character	Measure
Bizygomatic width	1,660
Estimated postorbital width	1,760
Width of occipital condyles	290
Distance between lateral margins of exoccipitals	850
Length of supraoccipital shield from foramen magnum to vertex	560
Height between basicranium and vertex	71
Transverse width of maxillae at vertex	290

**Note:**

Characters are measured as preserved.

**Rostrum:** Only a fragment of the right maxilla is preserved showing the typical transverse compression present in Balaenidae.

**Figure 6:**
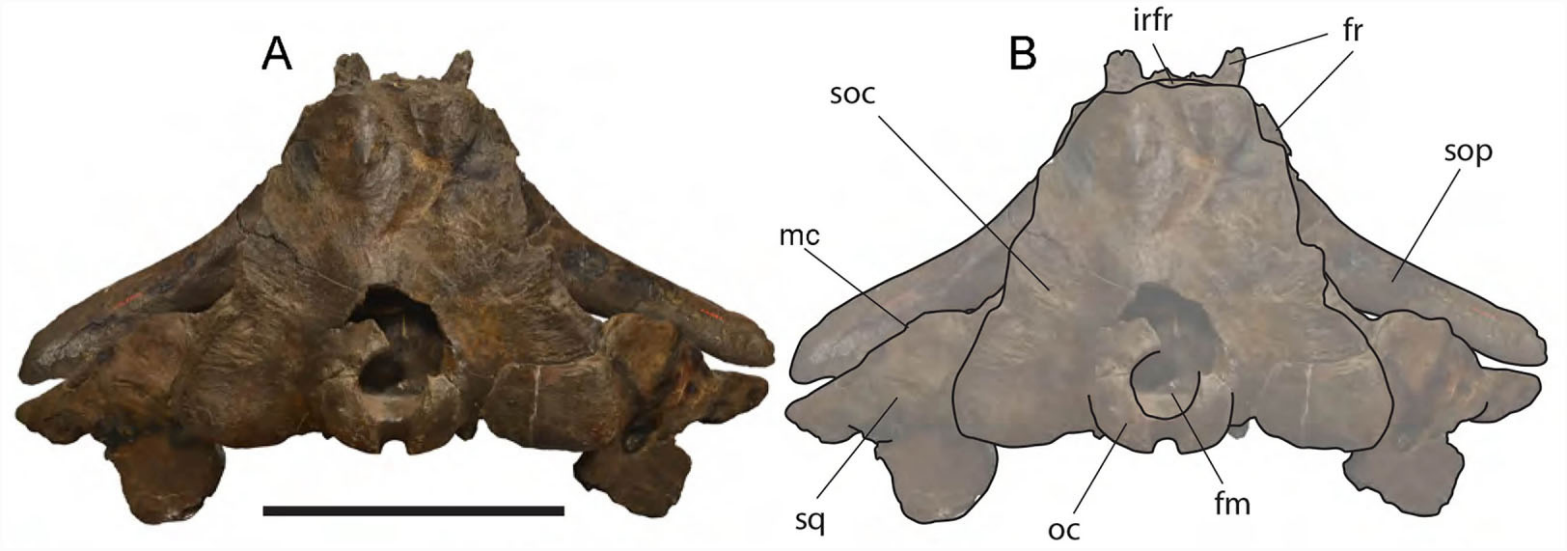
*Eubalaena ianitrix* sp. nov. (holotype RBINS M. 879). Dorsal view of neurocranium. (A) Photographic representation, (B) interpretation. Scale bar equals 50 cm. Anatomical abbreviations: fm, foramen magnum; fr, frontal; irfr, interorbital region of the frontal; oc, occipital condyles; smc, supramastoid crest; sq, squamosal; sop, supraorbital process of the frontal.

**Frontal:** Due to the erosion of the anterior-most border of the supraoccipital, it is possible to observe a tiny portion of the interorbital region of the frontal in dorsal view (Fig. 6). Prior to the erosion of the supraoccipital, that portion was superimposed by the anterior portion of the supraoccipital and was not visible. Judging from what is preserved, the interorbital region of the frontal was less bent than the supraoccipital suggesting that, in lateral view, the posterior portion of the rostrum was nearly flat as seen in *Eubalaena glacialis*. The transverse diameter of the interorbital region (measured along the inferred position of the nasofrontal suture) is *c.* 240 mm.

**Figure 7:**
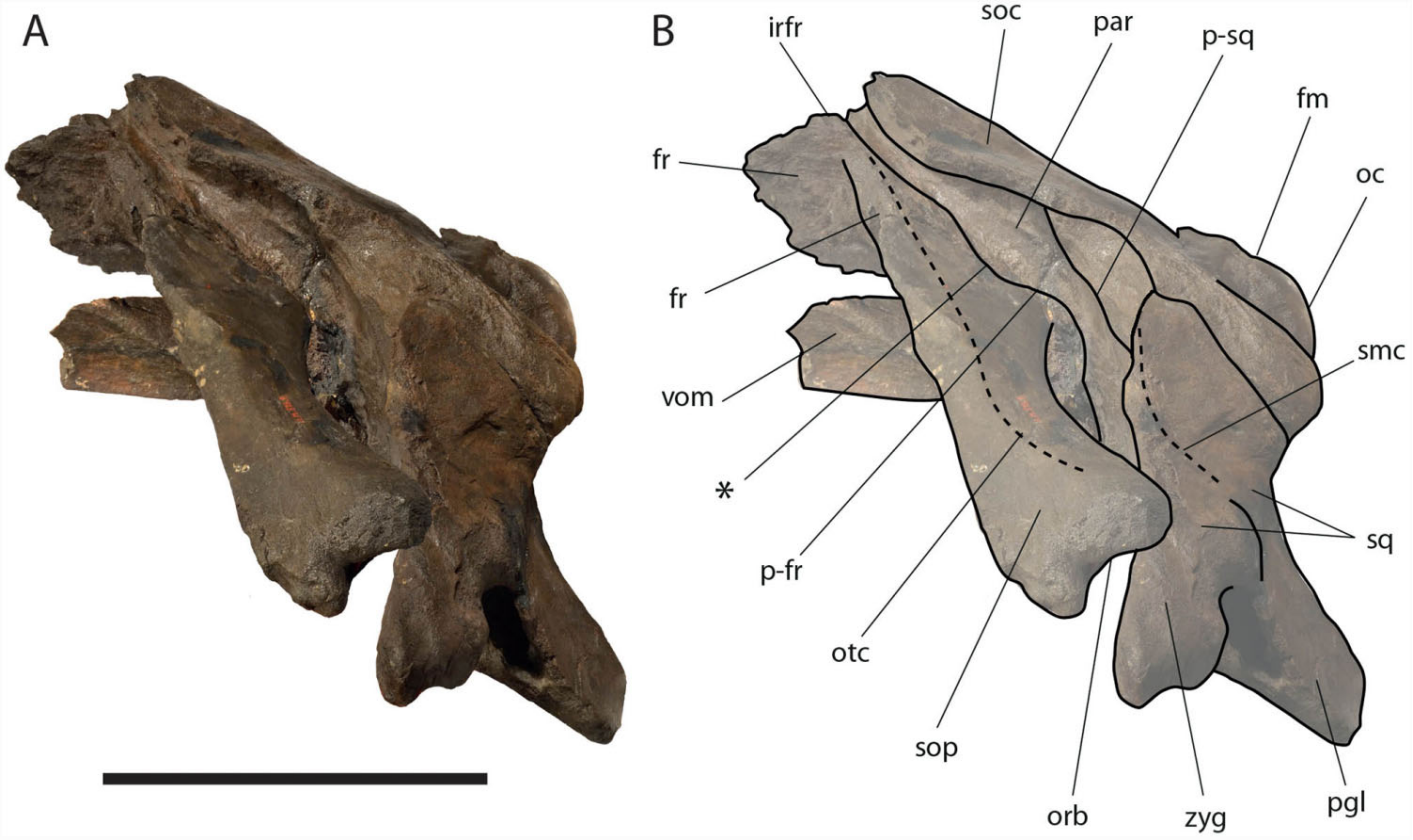
*Eubalaena ianitrix* sp. nov. (holotype RBINS M. 879). Left lateral view of neurocranium. (A) Photographic representation, (B) interpretation. Scale bar equals 50 cm. Anatomical abbreviations: fm, foramen magnum; fr, frontal; irfr, interorbital region of the frontal; oc, occipital condyle; orb, orbit; otc, orbitotemporal crest; par, parietal; pgl, postglenoid process of squamosal; p–fr, parietal–frontal suture; p–sq, parietal–squamosal suture; smc, supramastoid crest; soc, supraoccipital; sop, supraorbital process of frontal; sq, squamosal; vom, vomer; zyg, zygomatic process of squamosal; *, anterolateral corner of parietal–frontal suture.

**Figure 8:**
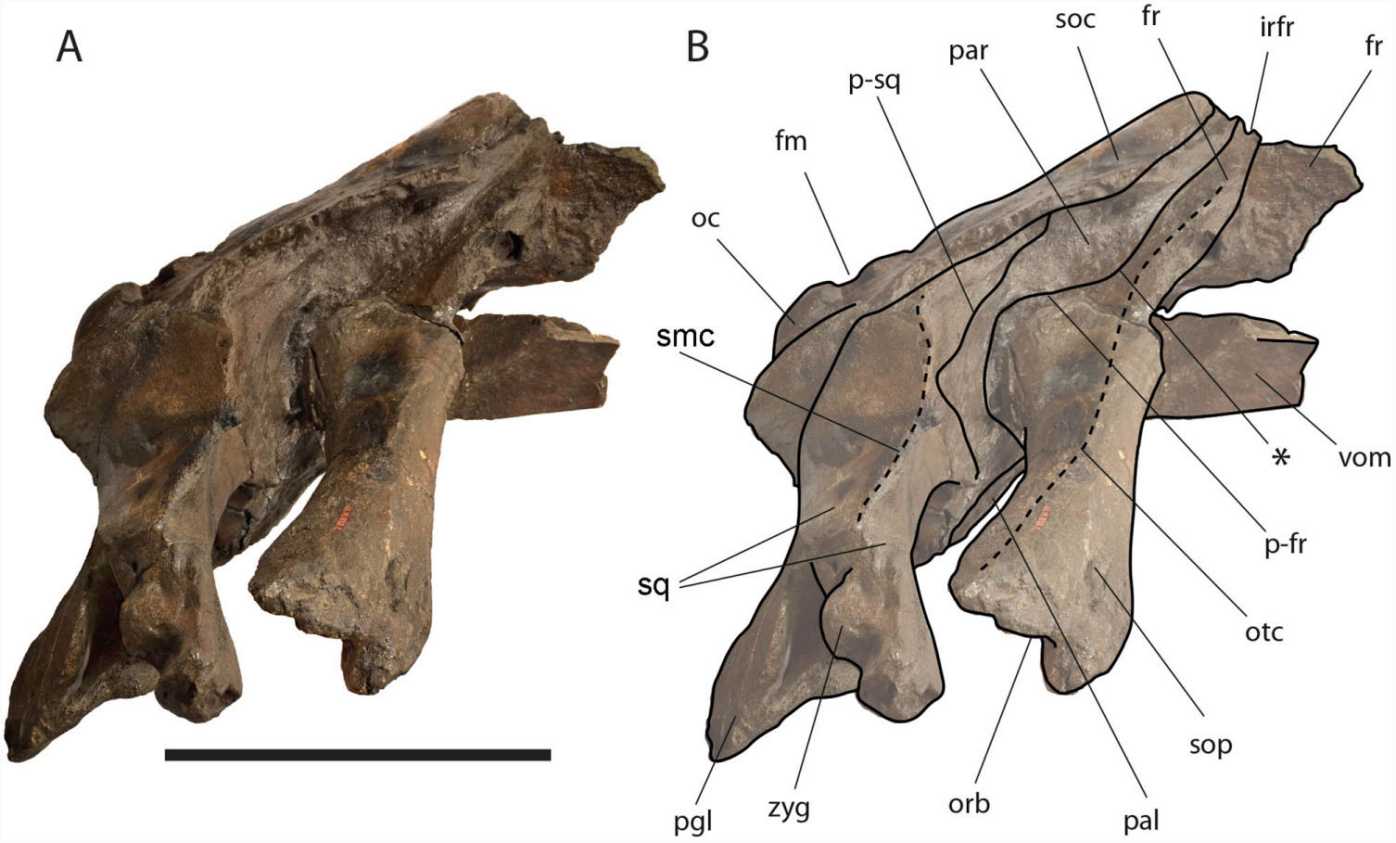
*Eubalaena ianitrix* sp. nov. (holotype RBINS M. 879). Right lateral view of neurocranium. (A) Photographic representation, (B) interpretation. Scale bar equals 50 cm. Anatomical abbreviations: fm, foramen magnum; fr, frontal; irfr, interorbital region of the frontal; oc, occipital condyle; orb, orbit; otc, orbitotemporal crest; par, parietal; pgl, postglenoid process of squamosal; p–fr, parietal–frontal suture; p–sq, parietal–squamosal suture; smc, supramastoid crest; soc, supraoccipital; sop, supraorbital process of frontal; sq, squamosal; vom, vomer; zyg, zygomatic process of squamosal; *, anterolateral corner of parietal–frontal suture.

**Figure 9:**
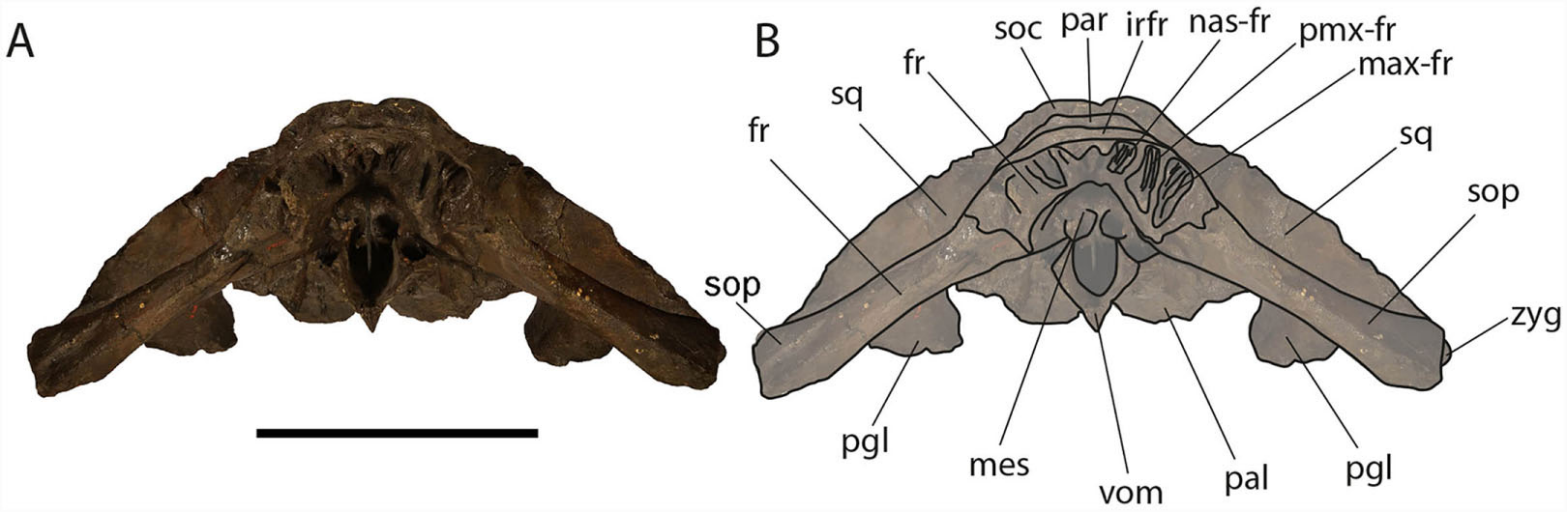
*Eubalaena ianitrix* sp. nov. (holotype RBINS M. 879). Anterior view of neurocranium. (A) Photographic representation, (B) interpretation. Scale bar equals 50 cm. Anatomical abbreviations: fr, frontal; irfr, interorbital region of the frontal; max–fr, grooves for articulation of maxilla and frontal; mes, mesethmoid; nas–fr, groove for articulation of nasal and frontal; pal, palatine; par, parietal; pgl, postglenoid process of squamosal; pm–fr, grooves for articulation of premaxilla and frontal; soc, supraoccipital; sop, supraorbital process of frontal; sq, squamosal; vom, vomer; zyg, zygomatic process of squamosal.

The supraorbital processes of the frontal are detached from the skull probably because post-mortem damage. The supraorbital process of the frontal is anteroposteriorly narrow and bears an evident but rounded orbitotemporal crest developed from the postorbital process to its anteromedial border (Figs. 6–8). The orbitotemporal crest is sharper proximally and becomes lower approaching the orbital rim. The right supraorbital process of the frontal is 650 mm in length up to the center of the orbit. The left supraorbital process of the frontal is 712 mm in length. A long groove for articulation with the maxilla is located at the anteromedial corner of the left supraorbital process of the frontal (Fig. 9).

**Figure 10:**
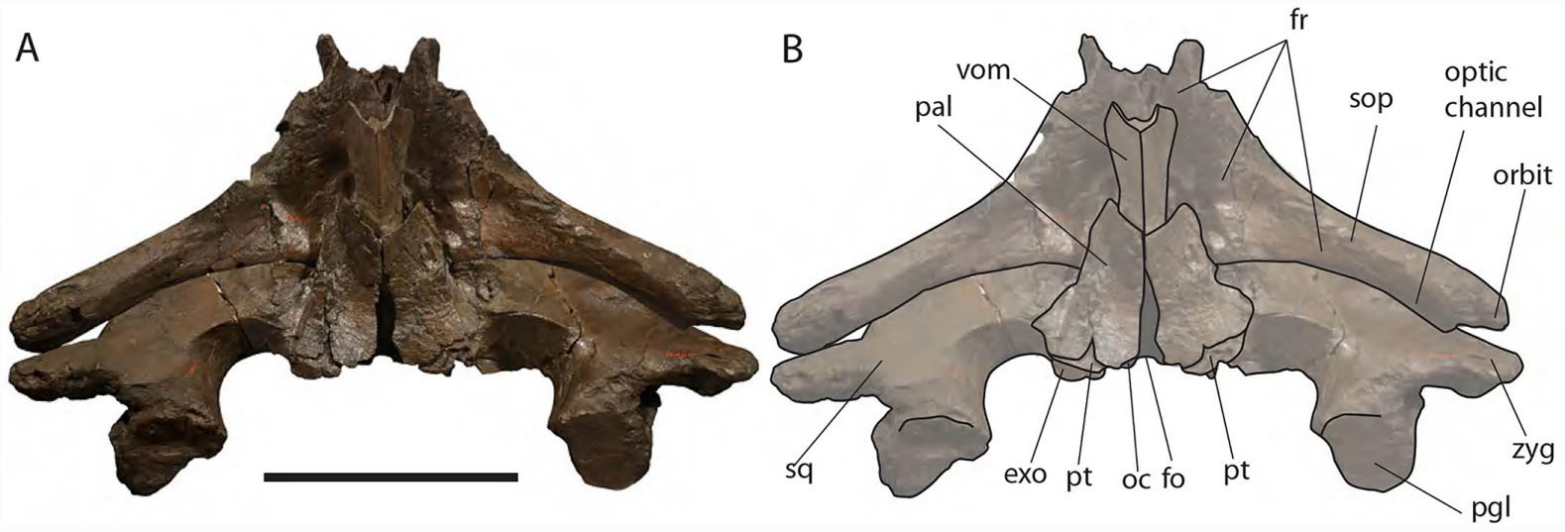
*Eubalaena ianitrix* sp. nov. (holotype RBINS M. 879). Ventral view of neurocranium. (A) Photographic representation, (B) interpretation. Scale bar equals 50 cm. Anatomical abbreviations: exo, exoccipital; fm, foramen magnum; fr, frontal; sop, supraorbital process of frontal; oc, occipital condyle; och, optic channel; or, orbit; pgl, postglenoid process of squamosal; pt, pterygoid; sq, squamosal; vom, vomer; zyg, zygomatic process of squamosal.

The optic canal is deep proximally (depth is *c.* 45 mm) and shallow distally (depth is *c.* 35 mm). Proximally, the right optic canal is bordered by anterior and posterior crests whose distance is 50 mm proximally and *c.* 100 mm distally (Fig. 10). The anteroposterior diameter of the left optic canal is 30 mm proximally at a distance of 400 mm from the orbital rim and 70 mm a few mm from the orbital rim.

Approaching the orbit, the dorsal surface of the supraorbital process of the frontal flattens. The right orbit is 170 mm in length (from the center of the postorbital process of the frontal to the center of the antorbital process of the frontal) and 51 mm in height (measured from the center of the orbital rim to an imaginary line joining antorbital and postorbital processes of the frontal). On the right side, antorbital and postorbital processes are similar in size but on the left side, the postorbital process is more robust than the antorbital process (Figs. 7 and 8). The longitudinal axis of the supraorbital process of the frontal is perpendicular to the imaginary line joining antorbital and postorbital processes. This suggests that, in the living animal, the supraorbital process of the frontal formed an approximately right angle with the lateral process of the maxilla and, thus, resembling the condition observed in the right whale of the genus *Eubalaena* and the fossil *Balaenula*.

The frontal of *Eubalaena ianitrix* shares the following characters with the living *Eubalaena* and *Balaenula*: presence of an evident orbitotemporal crest developed from the postorbital process to the anteromedial corner of the supraorbital process of the frontal, lack of dorsoventral compression along most of the length of the supraorbital process of the frontal (as seen in *M. parvus*, *Balaena mysticetus* and *Balaenella brachyrhynus*), presence of a right angle between supraorbital process of the frontal and the lateral process of the maxilla in lateral view, interorbital region of the frontal clearly angled with respect to the dorsoventral inclination of the supraoccipital. The articular groove for the maxilla combined with the short anteroposterior diameter of the proximal portion of the supraorbital process suggests that the ascending process of the maxilla was short and wide like that typically observed in the other Balaenoidea where this structure has been described (Bisconti, 2012 and literature therein). The short exposure of the interorbital region of the frontal on the dorsal surface of the skull and the exclusion of the parietal from exposure at cranial vertex are typical characters of living and fossil Balaenoidea.

**Parietal:** The parietal is evident on the lateral sides of the skull and at the cranial vertex due to the erosion of the anterior-most border of the supraoccipital (Fig. 6). Originally, the parietal was covered by the anterior border of the supraoccipital forming the nuchal crest. The frontal border of the parietal is superimposed on the interorbital region of the frontal obliterating it in dorsal view. More laterally, the frontal border descends ventrally and posteriorly and borders the posterodorsal portion of the supraorbital process of the frontal and forming an anteriorly convex coronal suture. Posteriorly to the supraorbital process of the frontal, the coronal suture forms a curve with anterior concavity and projects ventrally and posteriorly (Figs. 7 and 8).

The shape of the coronal suture is different in different balaenoid lineages. In the skull of *Caperea marginata* as seen in lateral view, the frontal border of the parietal gently descends from an anterodorsal point to a point located posterventrally in a straight-to-slightly convex line located dorsally to the supraorbital process of the frontal. This shape of the frontal border of the parietal is shared also with *Balaena mysticetes*, *Balaena montalionis*, *Balaena ricei* and *Balaenella brachyrhynus*. In the fossil *Miocaperea pulchra*, the right parietal shows a slightly different condition; in this species a distinctive anteroventral corner is located along the frontal border of the parietal (Bisconti, 2012). The anteroventral corner is present also in the species belonging to *Balaenula* and *Eubalaena* and in *Eubalaena ianitrix* (Figs. 7 and 8). In *Eubalaena australis*, posterior to the anteroventral corner, the frontal border shows a strong ventral concavity and a rounded shape making it distinct from the parietal of all the other balaenoid species.

The supraoccipital border of the parietal protrudes laterally and, together with the lateral border of the supraoccipital, forms the temporal crest. The temporal crest protrudes laterally and forms a sort of short roof of the temporal fossa in such a way that it prevents the medial wall of the temporal fossa (formed by the external surface of the parietal) from being observed in dorsal view. The external surface of the parietal is widely concave. Along the anteroposterior axis of the skull, the parietal appears short and high. The dorsal portion of the squamous border is anteroposteriorly elongated and bears a weak crest; the ventral portion of the squamous border forms a highly interdigitated suture with the squamosal and projects ventrally.

Among Balaenidae, a crest along the squamous border has been detected as a synapomorphy of *Balaena* and *Balaenella* by Bisconti (2005a) and Churchill, Berta & Deméré (2012) as it is absent from *Balaenula* and *Eubalaena*. It is not clear whether this crest is present in *Morenocetus* and *Peripolocetus*. The shape of the frontal border of the parietal differs from that observed in *Balaena* and *Balaenella* as it shows an undulating development; in *Balaena* and *Balaenella* the frontal border of the parietal proceeds posteroventrally as a straight line. A highly interdigitated ventral portion of the squamous border of the parietal is also observed in a subadult individual of *Eubalaena australis* (specimen NBC RGM 24757).

The squamous border of the parietal has distinctive characters in different balaenoid lineages. In *Caperea marginata*, the dorsal portion of the squamous border projects posteriorly to meet the supraoccipital (Bisconti, 2012). This character is also observed in *Balaena mysticetus* adult NBC RGM 373 and foetal NBC RGM 31116; the character was also illustrated by Cuvier (1823) (see Bisconti, 2003 for an image, *Eubalaena australis* adult IZIKO 2284, subadult NBC RGM 24757 and foetal IZIKO ZM 38950) and in the Pliocene *Eubalaena* sp. from Tuscany (Bisconti, 2002). In *Miocaperea pulchra* and *Balaenella brachyrhynus* the dorsal portion of the squamous border is nearly vertical. In *Eubalaena glacialis*, *Eubalaena japonica*, *Balaenula astensis* and *Eubalaena ianitrix* the dorsal portion of the squamous border projects anteriorly forming a finger-like structure that is deeply wedged between the parietal and the supraoccipital.

**Supraoccipital:** The supraoccipital is strongly built and represents the largest bone of this skull (Fig. 6). Parts of the anterior and lateral borders are missing due to post-mortem erosion of the skull and to damage done during the collection and preparation of the skull. The supraoccipital is wide and, as preserved, shows a convex lateral border and a widely rounded anterior border. The anteroposterior length (from the anterior border to the inferred position of the dorsal edge of the foramen magnum) is *c.* 531 mm; the transverse diameter is *c.* 350 mm anteriorly and *c.* 590 mm at mid-length. The external occipital protuberance, located on the anterior surface of the supraoccipital, is dorsally convex and forms a wide dome bordered by bilateral fossae located near the lateral borders of the supraoccipital. The dome consists of relief posteriorly subdivided by the interposition of a triangular, parasagittal fossa. There is a low sagittal crest located posteriorly to the dome. In lateral view, the dome is clearly visible as it protrudes dorsally and is not obliterated to view by the temporal crests. Before the post-mortem erosion of the skull, the supraoccipital formed a dorsal roof to the temporal fossa preventing the parietal from being observed in dorsal view.

In the genus *Eubalaena*, the supraoccipital is anteriorly wide and rounded and displays an external occipital protuberance that is dome-shaped. These characteristics of the supraoccipital are observed in all the living *Eubalaena* species, in the fossil *Eubalaena shinshuensis* and in the *Eubalaena* sp. described by Bisconti (2002) from the Pliocene of Tuscany. Subtle differences in the characters of the dome could be used for differentiating the species of *Eubalaena* but it is not completely clear whether the differences are due to individual variation or have taxonomic value. Bisconti (2002) described a sagittal crest on the external occipital protuberance and a series of five parasagittal crests posterior to it in a Pliocene *Eubalaena* sp. The five parasagittal crests are not observed in other *Eubalaena* species. A single sagittal crest is present in *Eubalaena australis* (NBC RGM 24757), *Eubalaena glacialis* (AMNH 42752, MSNT 264) *Eubalaena japonica* (Omura, 1958) and *Eubalaena ianitrix*.

The external supraoccipital protuberance is formed by a bilateral bulge in *Eubalaena australis* (NBC 24757), *Eubalaena glacialis* (AMNH 42752), *Eubalaena* sp. (Bisconti, 2002), and *Eubalaena ianitrix*, and by a single axial bulge in *Eubalaena japonica* and *Eubalaena shinshuensis* (Kimura, 2009). The external supraoccipital protuberance is a single bulge also in *Balaena mysticetus*, *Balaena montalionis*, *Balaena ricei* and *Balaenella brachyrhynus* but in these species the anterior portion of the supraoccipital is transversely constricted while in the species belonging to *Morenocetus*, *Balaenula* and *Eubalaena* the anterior portion of the supraoccipital is transversely wide.

Observations on skulls belonging to living species suggest that the lateral borders of the supraoccipital potentially undergo morphological change during ontogeny. In *Eubalaena australis*, the lateral border of the supraoccipital is externally convex in fetal and subadult individuals (ISAM ZM 38950, NBC RGM 24757). Omura (1958) observed that in adult individuals of *Eubalaena glacialis* the lateral border of the supraoccipital is more concave that in *Eubalaena japonica*. However, in the images provided by True (1904), an adult individual of *Eubalaena glacialis* has a continuously convex lateral border of the supraoccipital. It is possible that Omura’s (1958) observation was related to differences in the point of view from which the skulls were observed (Yamada et al., 2006).

**Vertex:** Based on Mead & Fordyce (2009, and literature therein) terminology, the vertex is the highest portion of the skull. In mysticetes it is formed by a mosaic of bones including supraoccipital, parietal, frontal and some posteromedial elements of the rostrum nasal and the ascending process of the premaxilla and of the maxilla. In *Eubalaena ianitrix*, the supraoccipital overlaps onto the parietal and prevents it from being observed in dorsal view (Fig. 6). The parietal is superimposed onto the interorbital region of the frontal that is, thus, scarcely visible in dorsal view. The only portion of the interorbital region of the frontal that can be observed is that that is immediately posterior to the nasofrontal suture. Judging from the articular groove present on the anteromedial surface of the supraorbital process of the frontal, the ascending process of the maxilla had a limited posterior extension resembling other living and fossil Balaenoidea.

The supraoccipital superimposition on the parietal and the parietal superimposition on the interorbital region of the frontal are synapomorphies of Balaenidae and Neobalaenidae and are not shared with other mysticete taxa (Bisconti, 2012 and literature therein). The lack of parietal exposure at the cranial vertex is another exclusive feature of Balaenidae and Neobalaenidae and is observed in all the living and fossil taxa belonging to these groups (Churchill, Berta & Deméré, 2012; Bisconti, 2003).

**Figure 11:**
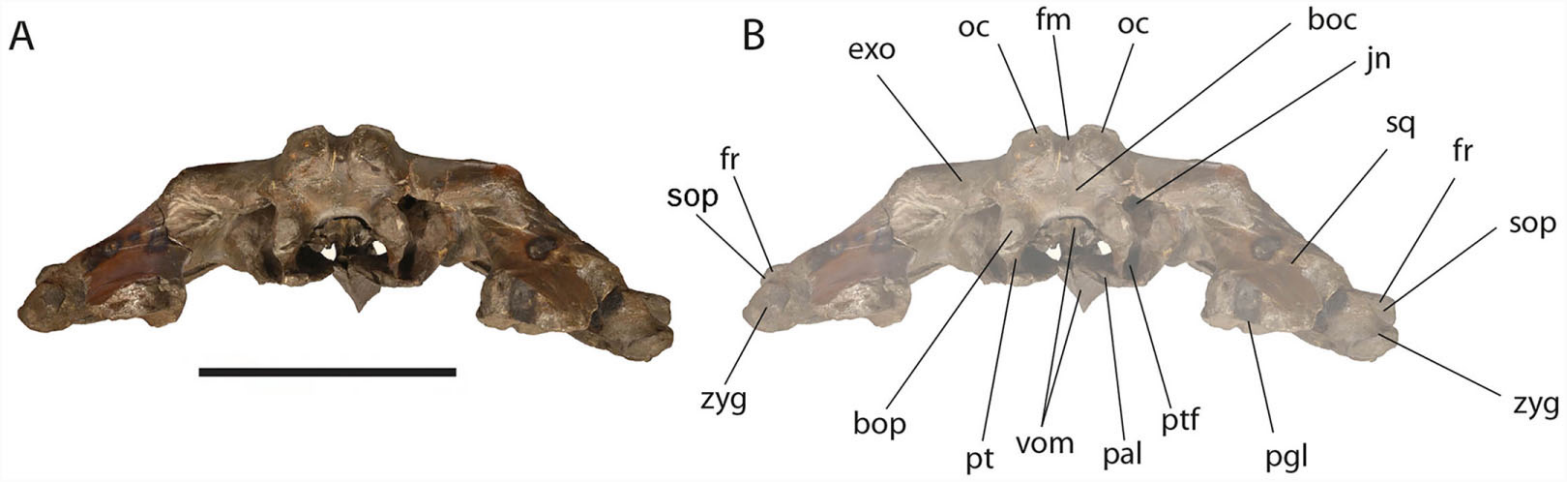
*Eubalaena ianitrix* sp. nov. (holotype RBINS M. 879). Posterior view of neurocranium. (A) Photographic representation, (B) interpretation. Scale bar equals 50 cm. Anatomical abbreviations: boc, basioccipital; bop, basioccipital protuberance; exo, exoccipital; fm, foramen magnum; fr, frontal; jn, jugular notch; oc, occipital condyle; pal, palatine; pgl, postglenoid process of squamosal; pt, pterygoid; ptf, pterygoid fossa; sop, supraorbital process of frontal; sq, squamosal; vom, vomer; zyg, zygomatic process of squamosal.

**Exoccipital:** The lateral portion of the exoccipital is a wide and flat surface with external border squared (Fig. 11). Only the left paroccipital process is preserved and appears strong and rugose in ventral view. A squared external border of the exoccipital is observed in *Eubalaena japonica* and, at a lesser extent, in *Eubalaena australis*. In *Eubalaena glacialis* the external border has a rounder shape than in those species. In *Balaena mysticetus* and *Balaena montalionis* the external border of the exoccipital appears anterolaterally round with a distinctive lateroventral corner that is observed also in *Eubalaena glacialis* but that seems absent in *Eubalaena japonica* (Omura, 1958). In lateral view, the exoccipital has a squared shape in *Eubalaena glacialis*, *Eubalaena australis*, *Eubalaena japonica* and the species belonging to *Balaenula* but it is not clear whether a squared shape is also present in *Eubalaena ianitrix*.

The occipital condyle is wide, reniform and its surface for articulation with the atlas is nearly flat along both the dorsoventral and the lateromedial axes. The main axis of the occipital condyle is oriented from a posteroventral point to an anterolateral point. There is a wide intercondyloid fossa located ventrally between the condyles. The condyles are not in contact each other ventrally or dorsally. The maximum anteroposterior diameter of the occipital condyle is 190 on the right side and 170 on the left side; the maximum lateromedial diameter of the occipital condyle is 101 on the right side and 107 on the right side. The condyles surround a wide foramen magnum whose dorsal border is not preserved. The maximum tansverse diameter of the foramen magnum is 145 mm and its dorsoventral diameter is inferred to be *c.* 140 mm based on a nearly circular outline with a slight dorsoventral compression as seen in other balaenid species. The distance between the external borders of the occipital condyles is *c.* 350 mm.

**Squamosal:** Right and left squamosals are partly broken; breakage lines are straight enough to allow an easy reconstruction of this part of the skull by putting the broken portions of the squamosals in place through right connections (Figs. 7 and 8).

The parietal margin of the squamosal forms the squamosal-parietal suture. Dorsally, this suture projects anteriorly making it possible for the squamosal to be deeply inserted between the supraoccipital and the parietal. More ventrally, the squamosal-parietal suture is highly interdigitated.

The squamosal plate is dorsoventrally and anteroposteriorly concave and, in lateral view, it is hidden by the anterior and ventral development of an anteriorly convex supramastoid crest. The supramastoid crest is protruding anterolaterally and shows a widely rounded anterior shape. The supramastoid crest is separated from the zygomatic process of the squamosal by a wide anterior concavity. The zygomatic process of the squamosal is short and stocky; its main axis projects laterally and ventrally in dorsal view.

The squamosal has a clear dorsoventral development as typically observed in Balaenidae. Its dorsoventral diameter is 550 mm on the external surface (from the exoccipital-squamosal suture to the anterior end of the zygomatic process of the squamosal) of the right squamosal. The glenoid fossa of the squamosal is largely eroded; what remains suggests that it was flat or scarcely concave as seen in other typical balaenid whales. The glenoid fossa of the right squamosal is 470 mm in anteroposterior length.

Posterodorsally, the site for the articulation with the posterior process of the petrotympanic is developed ventrally to the exoccipital–squamosal suture and is ventrally bordered by a crest that separates it from the external acoustic meatus. Both this site and the external acoustic meatus are represented by transverse and tube-like concavities developed along the dorsal and posterior portion of the squamosal. The posterior border of the foramen ovale is made of the squamosal and the pterygoid.

The squamosal of *Eubalaena ianitrix* shows the following typical balaenid characters: dorsoventral elongation, reduction of the zygomatic process of the squamosal, scarcely concave glenoid fossa of the squamosal, widely rounded supramastoid crest in lateral view. In *Balaenella* and in the species of *Balaena* the squamosal is also posteroventrally oriented (Bisconti, 2000) but this character is not observed in *Eubalaena ianitrix*. Rather, the squamosal of *Eubalaena ianitrix* appears more vertical resembling *Morenocetus*, *Balaenula* and the living species of *Eubalaena*. In *Balaenella brachyrhynus*, *Balaena mysticetus*, *Balaena ricei* and *Balaena montalionis* the zygomatic process of the squamosal projects more laterally allowing the view of the posterior wall of the temporal fossa formed by the squamosal plate. In *Eubalaena*, *Balaenula* and *Eubalaena ianitrix* this is not the case as the zygomatic process of the squamosal projects anteriorly and prevents the posterior wall of the temporal fossa from being observed in lateral view.

**Alisphenoid:** The alisphenoid is exposed in the temporal fossa. It has a triangular shape. It is bordered anteriorly by the supraorbital process of the maxilla, ventrally by the palatine, and dorsally and posteriorly by the squamosal.

The alisphenoid is exposed in the temporal fossa in *Eubalaena glacialis* and *Eubalaena japonica* but it is not clear whether such an exposure occurs also in *Eubalaena australis*. In fetal specimen (IZIKO ZM 38950) the alisphenoid is observed in the temporal fossa but in subadult individual (NBC RGM 24757) the alisphenoid is only visible in ventral view and does not appear in the temporal fossa as the ventral border of the squamosal superimposes onto it. In *Balaena mysticetus*, *Balaena brachyrhynus* and in the genus *Balaenula* the alisphenoid is inferred to be exposed in the temporal fossa based on the articular pattern of squamosal and parietal. The alisphenoid was originally bordered by the squamosal dorsally and posteriorly and by the parietal dorsally and anteriorly, by the palatine ventrally.

**Temporal fossa:** The temporal fossa of *Eubalaena ianitrix* is dorsally overhung by the lateral projection of the temporal crest formed by the lateral border of the supraoccipital and the dorsal border of the parietal (Fig. 6). The lateral extension of the temporal crest is difficult to assess because the lateral edge of the supraoccipital and the dorsal border of the parietal are damaged. The medial wall of the temporal fossa is formed by parietal, squamosal and alisphenoid. The alisphenoid is not in contact with the parietal; the parietal–squamosal suture is highly interdigitated ventrally but, dorsally, the squamosal forms a digit-like anterior protrusion that is deeply inserted between the supraoccipital and the parietal. The medial wall of the temporal fossa is concave both dorsoventrally and anteroposteriorly. The posterior wall of the temporal fossa is formed by the squamosal and shows an anterior concavity. Lateral to the posterior wall of the temporal fossa, the supramastoid crest protrudes anteriorly and forms the lateral border of the squamosal fossa.

The general features of the temporal fossa of *Eubalaena ianitrix* are also observed in *Eubalaena glacialis* and *Eubalaena japonica. Eubalaena australis* differs in the lack of exposure of the alisphenoid in the temporal fossa at adulthood. In the Pliocene *Eubalaena* sp. from Tuscany (Bisconti, 2002) and *Eubalaena shinshuensis* (Kimura, 2009) the digit-like projection of the anterodorsal portion of the squamosal is absent. In *Balaenula* the posterior apex of the lambdoid crest is located much more anteriorly than in any species belonging to *Eubalaena*, *Balaena* and *Balaenella* and this makes its temporal fossa anteroposteriorly smaller; moreover, in *Balaenula astensis* the posterior wall of the temporal fossa is mainly flat along the dorsoventral axis (Bisconti, 2000, 2003).

**Palatine:** The palatine is almost rectangular in ventral view (Fig. 10). It is an elongated bone that is anteriorly in contact with the maxilla and posteriorly with the pterygoid. As typically observed in Balaenidae, the palatine is ventrally superimposed on the ventral lamina of the pterygoid that appears, in ventral view, as a small stripe of bone close to the posterior limit of the skull. The ventral surface of the palatine is almost flat. The longitudinal axis of the palatine diverges from the anteroposterior axis of the skull posteriorly as the posterior ends of the palatines are not in contact posteriorly. The lateral lamina of the palatine ascends and contacts the squamosal, the alisphenoid and the frontal.

The relationships of the palatine observed in *Eubalaena ianitrix* are not different from those that can be observed or inferred in other living and fossil Balaenidae for which information about this bone is available.

**Pterygoid:** Following Churchill, Berta & Deméré (2012), Bisconti (2000, 2005a) and Fraser & Purves (1960), in Balaenidae the pterygoid appears as a small stripe of bone in ventral view. This stripe of bone represents the lateral lamina of the pterygoid that is transversely elongated and approaches the posterior-most border of the skull in lateral view. The pterygoid is dorsally, anteriorly and posteriorly bordered by the squamosal and anteroventrally by the palatine. The posterior border of the pterygoid and the anterior border of the falciform process of the squamosal contribute to delimit the shape of the foramen ovale (Fig. 10).

Apart from *Caperea marginata*, in which the foramen ovale is within the pterygoid, the foramen ovale of other balaenoids is located between the squamosal and the pterygoid. In the living species the foramen ovale extends into a tube formed almost entirely by the squamosal (= infundibulum of Fraser & Purves, 1960). This condition is not observed in *Eubalaena ianitrix* where the foramen ovale has an elliptical shape.

### Body size estimate

Two of the chosen methods converge toward a total body length or *c.* 6–8 m. The application of Eq. (1) based on a bizygomatic width of 1,660 mm (Table 2) retrieved a total body length of *c.* 13 m; this result is to be corrected by reducing it of 37-to-47%. After the correction, the resulting values are respectively *c.* 8 m and *c.* 7 m.

The application of the regression Eq. (4) based on a supraoccipital length of 560 mm (Table 2) found a condylobasal length of *c.* 1.6 m. After having tripled and quadrupled this length, the total body length was estimated between 4.74 and 6.37 m.

The application of the Eq. (2) based on an occipital breadth of 353 mm retrieved a body mass of *c.* 33 t. This value is consistent with weight values obtained by Omura (1958) for the North Pacific right whale (*Eubalaena japonica*). We used this body mass estimate in the Eq. (3) and found a total body length of *c.* 11 m, which is closer to the result obtained from the Eq. (1) before the correction. It is not clear whether the results of the Eq. (3) need to be corrected but, following the suggestions of Pyenson & Sponberg (2011), we hypothesize that a correction would be necessary that should be around 40%. If we apply such a correction to the value obtained by the Eq. (3), we find a total body length of *c.* 6.6 m that is very close to the higher results of the Eqs. (1) and (4). If we accept a total body length between 6 and 7 m then we need to apply a roughly similar correction to the estimated body weight. If we reduce the estimated body weight of 40% then we obtain an estimated body weight of 19.8 t.

We therefore estimate the total body length of the holotype specimen of *Eubalaena ianitrix* between 5 and 7 m, with a body mass of *c.* 20 t.

## Phylogeny

### Overview

**Figure 12:**
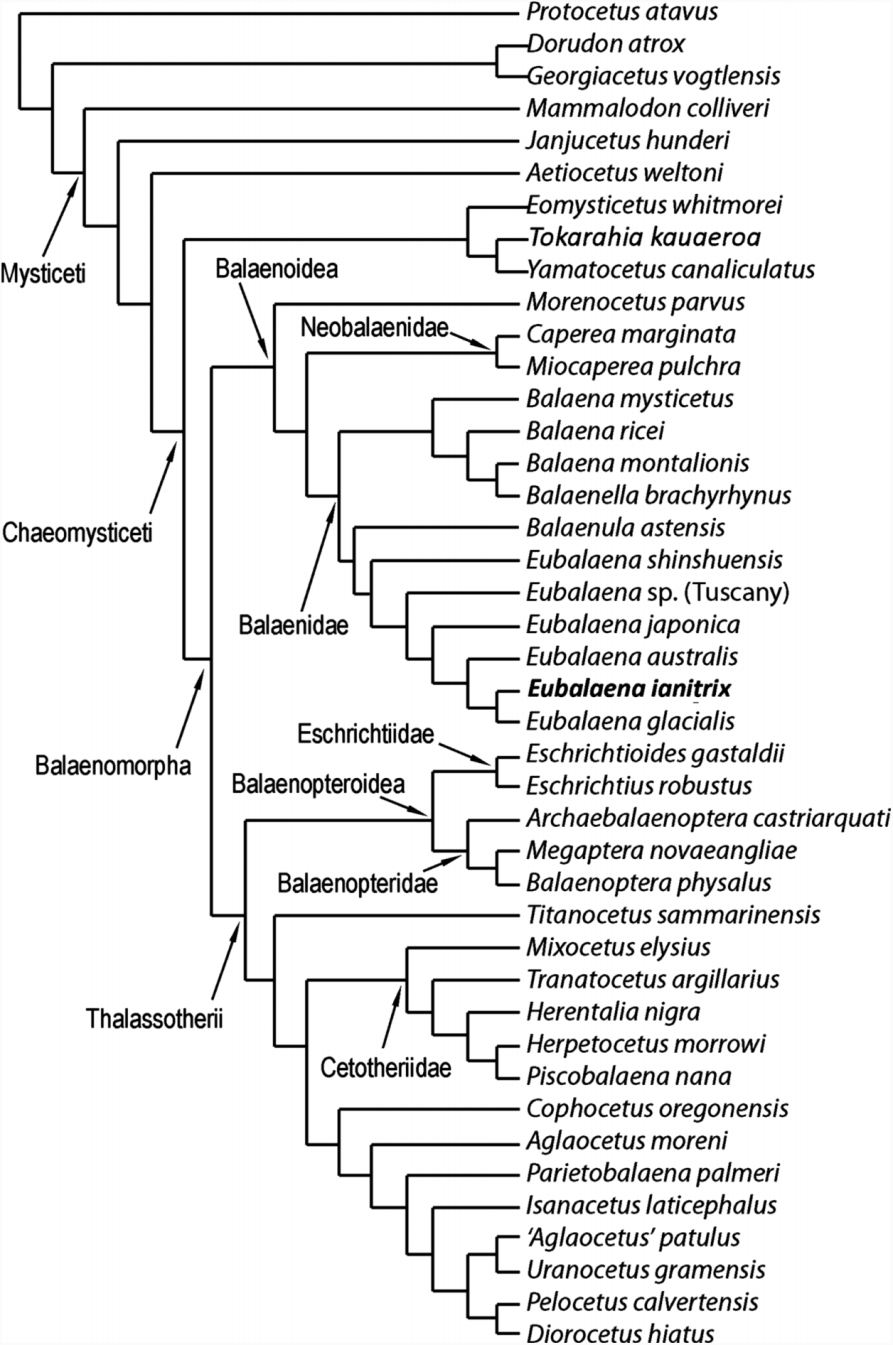
Phylogenetic relationships of Mysticeti with focus on Balaenoidea. Single most-parsimonious cladogram with the following tree statistics: consistency index (CI), 0.508; retention index (RI), 0.805; rescaled CI, 0.40894; homoplasy index (HI), 0.492; stratigraphic consistency index (SCI), 0.825.

The phylogenetic analysis resulted in the single most parsimonious cladogram shown in Fig. 12. Tree statistics are provided in the corresponding caption. Our results confirm the monophyly of Mysticeti, Chaeomysticeti and Balaenomorpha. The sister-group of Balaenomorpha is the monophyletic Eomysticetidae (here represented by *Eomysticetus whitmorei*, *Tokaraia kauaeroa* and *Yamatocetus canaliculatus*). Balaenomorpha is then subdivided into two sister-groups: Balaenoidea and Thalassotherii (including Balaenopteridae, Eschrichtiidae, Cetotheriidae and basal thalassotherian taxa including *Cophocetus*, *Aglaocetus*, *Parietobalaena*, *Isanacetus*, *Uranocetus*, *Pelocetus* and *Diorocetus*). As such, the present results confirm the monophyly of Balaenopteroidea (including Balaenopteridae and Eschrichtiidae) and Cetotheriidae (here including *Mixocetus*, *Herentalia*, *Piscobalaena*, *Herpetocetus* and *Tranatocetus*). *Tranatocetus argillarius* is nested here among Cetotheriidae. Although this may be due to our limited sample of Cetotheriidae and related taxa, we are unable to support the monophyly of Tranatocetidae (as proposed by Gol’din & Steeman (2015)), considering that *T. argillarius* (the only nominal Tranatocetidae taxon included in our analysis) falls within Cetotheriidae.

Most surprising are the position of *M. parvus* (that will be discussed in the next paragraph) and the sister-group relationships within Thalassotherii. Among Thalassotherii, four monophyletic groups of family-level rank are recognized: Balaenopteridae, Eschrichtiidae, Cetotheriidae and a clade including what Bisconti, Lambert & Bosselaers (2013) called basal thalassotherian taxa. Eschrichtiidae is the sister-group of Balaenopteridae and both form the monophyletic Balaenopteroidea. Balaenopteroidea is the sister-group of a large clade including *Titanocetus sammarinensis*, Cetotheriidae and basal thalassotherian taxa. *Ti. sammarinensis* is, in its turn, the sister-group of Cetotheriidae and basal thalassotherian taxa.

### Relationships of Balaenoidea and morphological support to nodes

Our results support the monophyly of Balaenoidea with a noticeable difference with respect to previously published literature (Cabrera, 1926; Bisconti, 2005; Churchill, Berta & Deméré, 2012): *M. parvus* falls outside Balaenidae + Neobalaenidae and represents the sister-group of both families.

Nine synapomorphies support the monophyly of Balaenoidea. Three of them depends on the structure of the skull: characters 37 (short exposure of interorbital region of the frontal because of superimposition by the parietal), 54 (massive elongation of supraoccipital) and 55 (supraoccipital is superimposed onto the interorbital region of the frontal). Moreover, character 47 (squamosal dorsoventrally elongated) is also an exclusive synapomorphy of this clade.

Seventeen synapomorphies support the monophyly of Neobalaenidae + Balaenidae to the exclusion of *M. parvus*. Three of them are unambiguous: characters 81 (short dorsoventral height of the tympanic cavity), 82 (dorsoventrally compressed tympanic bulla) and 83 (enlargement of epitympanic hiatus). Characters 11 (rostrum highly arched), 84 (anteroposteriorly short anterolateral lobe of tympanic bulla), 92 (dorsal exposure of mandibular condyle), 95 (dorsoventral arc of dentary along the whole length of the bone) and 101 (cervical vertebrae fused) represent additional ambiguous synapomorphies of the clade. Neobalaenidae (including *Caperea* and *Miocaperea*) is the sister-group of Balaenidae (here including *Balaena, Balaenella, Balaenula* and *Eubalaena*). The monophyly of Neobalaenidae is supported by four synapomorphies including a reversal in character 122 (complete infundibulum). Characters 50 (presence of squamosal cleft) and 75 (exposure of posterior process of petrotympanics in the lateral view of the skull) are ambiguous synapomorphies as these characters (in different ways) are observed in Balaenopteridae and Cetotheriidae, presumably as a result of convergent evolution.

Four unambiguous synapomorphies support the monophyly of Balaenidae: characters 64 (massive elongation of palatine posteriorly), 65 (posterior placement of pterygoid), 86 (sharply defined groove for mylohyoidal muscle) and 122 (foramen ovale with incomplete infundibulum). Three additional ambiguous synapomorphies are detected: characters 12 (transverse compression of maxilla), 74 (long and thick roof of stylomastoid fossa) and 97 (strong anterior torsion of dentary).

Balaenidae is subdivided into two clades: one including *Balaena* and *Balaenella* and the other including *Balaenula* and *Eubalaena*. The inclusion of *Balaenella brachyrhynus* within *Balaena* casts some taxonomic problems as it either makes *Balaena* paraphyletic or suggests inclusion of *Balaenella* within *Balaena. Balaenella brachyrhynus* and *Balaena montalionis* share an anteriorly narrowed supraoccipital and a supraoccipital with transversely short anterior border; these character states support their sister-group relationship. Unfortunately, a clear illustration of the dorsal view of *Balaena ricei* is not available and it is difficult to understand whether this species is really more closely related to *Balaena montalionis* and *Balaenella brachyrhynus* or to *Balaena mysticetus*. From our results, *Balaena mysticetus* represents a separate lineage that diverged before the other *Balaena*-like taxa (*Balaena ricei*, *Balaena montalionis* and *Balaenella*). A low number of synapomorphies support the monophyly of the clade including *Balaena* and *Balaenella*. These include the following two unambiguous synapomorphies: characters 116 (transverse compression of anterior supraoccipital) and 120 (lateral projection of zygomatic process of the squamosal). Additionally, two ambiguous synapomorphies are also found to support this clade; these include characters 126 (posterior orientation of dorsoventrally developed squamosal body) and 132 (crest present at parietal–squamosal suture). The sister-group relationship of *Balaena montalionis* and *Balaenella brachyrhynus* is supported by one unambiguous synapomorphy (character 117: squared anterior border of supraoccipital) and one ambiguous synapomorphy (character 118: short anterior border of supraoccipital).

### Relationships of *Eubalaena*

Confirming previously published hypotheses (Bisconti, 2000, 2005a; Churchill, Berta & Deméré, 2012), our analysis resulted in the monophyly of a clade including *Balaenula* and *Eubalaena* (Fig. 12). The clade including *Eubalaena* and *Balaenula* is the sister-group to the *Balaena* + *Balaenella* clade. *Balaenula* is the sister-group of *Eubalaena*. Three unambiguous and one ambiguous synapomorphies support this clade. The unambiguous synapomorphies include characters 123 (transverse orientation of supraorbital process of the frontal in lateral view), 129 (curvature of rostrum with horizontal proximal part) and 130 (concavity on the anterior border of nasal). Character 118 (transversely wide anterior border of supraoccipital) was also found to support this clade (ambiguous synapomorphy).

*Eubalaena shinshuensis* is the first *Eubalaena* species to branch; the *Eubalaena* sp. from the Late Pliocene of Tuscany is the sister-group of the living *Eubalaena* species + *Eubalaena ianitrix* and its inclusion on a separate ramus suggests that it could be a different *Eubalaena* species of its own. *Eubalaena japonica* and *Eubalaena australis* branch before *Eubalaena ianitrix* and *Eubalaena glacialis*, the two latter being sister-groups.

Only one unambiguous synapomorphy was found to support the monophyly of the right whale genus *Eubalaena*; character 115 (presence of a dome on the supraoccipital). We think that this reduced morphological support for the well-established *Eubalaena* genus is due to the fact that most of the characters previously used to support its monophyly are shared with *Balaenula. Eubalaena shinshuensis* from the Messinian of Japan was found to be the earliest-diverging right whale species of the genus; the Pliocene *Eubalaena* sp. from Tuscany is the sister-group of the living *Eubalaena* species + *Eubalaena ianitrix*. The monophyly of the *Eubalaena* sp. from Tuscany and the crownward *Eubalaena* species was supported by one unambiguous synapomorphy (character 127: squared exoccipital in lateral view) and one ambiguous synapomorphy (character 126: vertical orientation of squamosal body).

**Figure 13:**
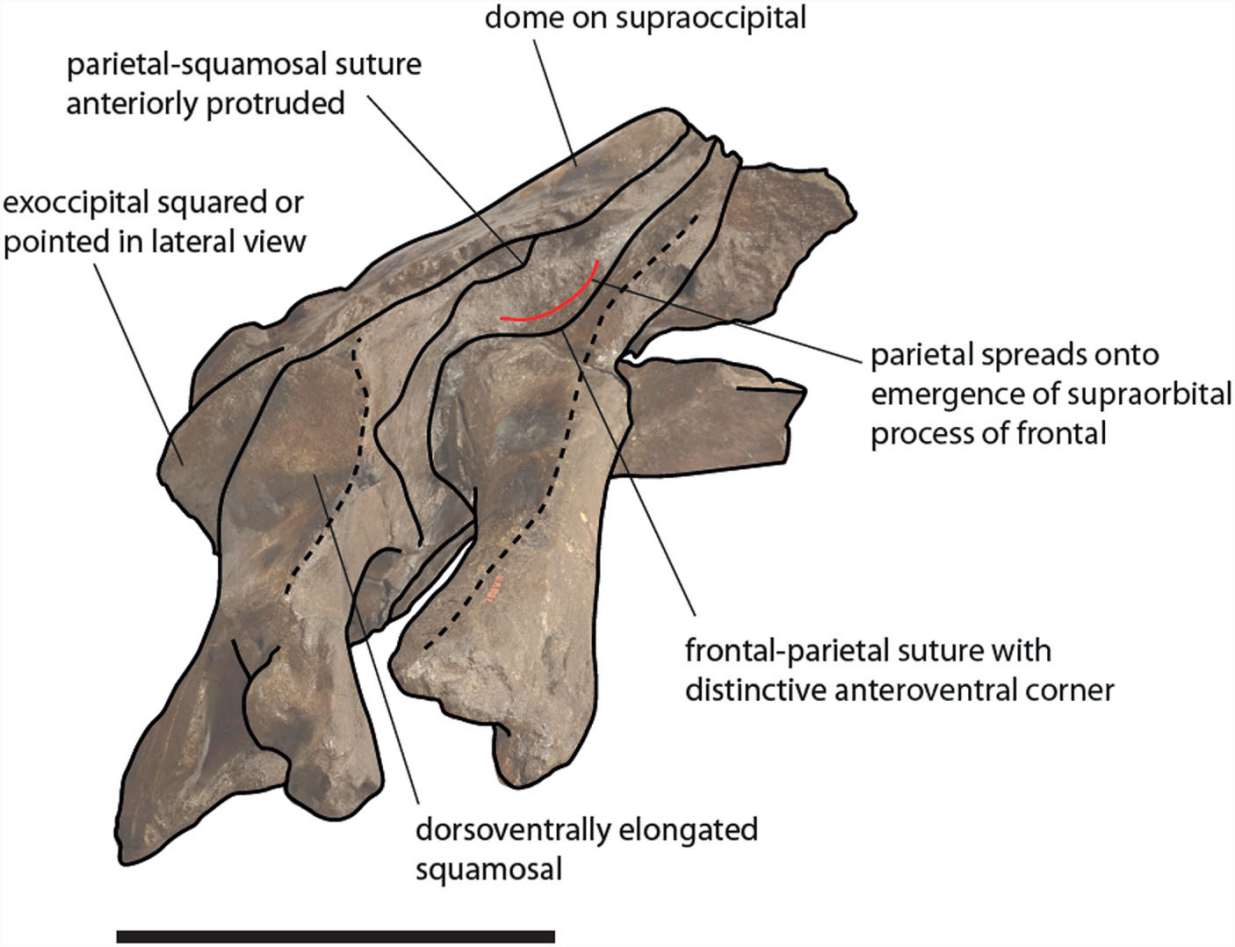
Schematic representation of diagnostic characters observed in the holotype skull of *Eubalaena ianitrix* in right lateral view. Not to scale.

The clade including the living *Eubalaena* species and *Eubalaena ianitrix* is supported by five unambiguous synapomorphies (125: parietal–frontal suture with distinctive anteroventral corner; 131: short nasals; 133: parietal spreads on the supraorbital process of the frontal; 140: presence of vascular groove on posterior part of pars cochlearis; and 141: evident pyramidal process posterior to perilymphatic foramen) and eight ambiguous synapomorphies (114: sagittal concavity on supraoccipital; 134: anterior protrusion of parietal–squamosal suture; 135: prismatic posterior process of petrosal; 138: transversely elongated pars cochlearis; 143: long transverse process of the atlas; 146: highly concave anterior and posterior borders of humerus; 147: globular humeral head; 150: superior corner of olecranon reduced-to-absent; and 151: reduced-to-absent coracoid process in scapula) (Fig. 13).

*Eubalaena australis* was found to be more closely related to *Eubalaena ianitrix* + *Eubalaena glacialis* than *Eubalaena japonica*. The sister-group relationship of *Eubalaena glacialis* with *Eubalaena ianitrix + Eubalaena glacialis* was supported by two unambiguous synapomorphies: characters 139 (crista transversa exits from internal acoustic meatus) and 152 (transverse orientation of tyrohyoidal processes). It is noticeable that none of these characters is preserved in the holotype of *Eubalaena ianitrix* and the placement of this species in this precise position in the cladogram relies on ACCTRAN optimization of the morphological transformations operated by TNT. The monophyly of the clade *Eubalaena ianitrix* + *Eubalaena glacialis* is supported by a single ambiguous synapomorphy: character 121 (presence of pterygoid in temporal fossa).

### Stratigraphic consistency index

The calculation of the stratigraphic consistency index shows that the degree of agreement of the branching pattern with the stratigraphic occurrence of the taxa is exceptionally high. The SCI depends on (1) the number of well-resolved nodes and (2) the number of stratigraphically consistent nodes. In the hypothesis of phylogeny presented in this paper, the maximum number of nodes is 40 (number of OTUs minus 2) and the number of stratigraphically consistent nodes is 33. The SCI is thus 0.825.

### Divergence dates of balaenoid clades

**Figure 14:**
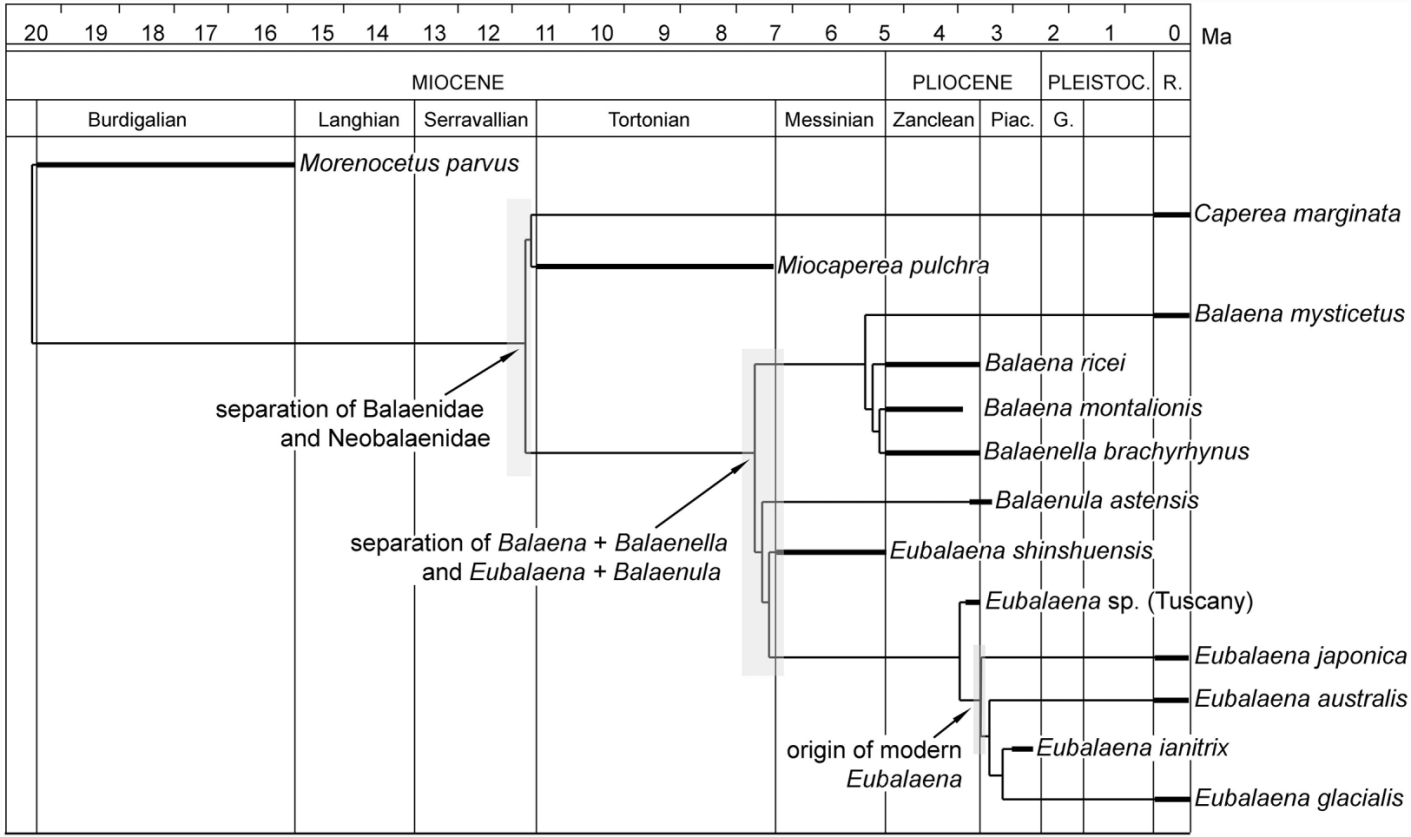
Phylogenetic relationships of Balaenidae plotted against temporal scale. Phylogenetic relationships of Balaenidae plotted against a temporal scale showing that the separation of Balaenidae and Neobalaenidae occurred at least before Tortonian and that the separation of the *Balaena + Balaenella* clade from the *Eubalaena + Balaenula* clade occurred at the beginning of the Messinian or during this stage (latest Miocene). The origin of the modern *Eubalaena* (right whale) is inferred to have occurred at the beginning of the Piacenzian.

In Fig. 14, the hypothesis of phylogeny for Balaenoidea proposed in the present paper is plotted against the stratigraphic age of the included OTUs. In the figure, branch lengths are inferred from the phylogenetic relationships of the taxa and from the stratigraphic ages of the representative fossil record of each OTU.

The age of *Miocaperea pulchra* suggests that the origin of the clade including Balaenidae + Neobalaenidae is older than Tortonian (early Late Miocene). Unfortunately, given that *M. parvus* falls outside Neobalaenidae + Balaenidae, it is not possible to be sure about the precise age of origin of these families. Indeed, as the stratigraphic occurrence of *M. parvus* is limited to the Burdigalian (late Early Miocene), the age of origin of Neobalaenidae and Balaenidae may be constrained to a time interval between Burdigalian and Tortonian.

The fossil record of *Balaena*-like species does not extend before Zanclean (Early Pliocene). The stratigraphic occurrences of *Balena montalionis*, *Balena ricei* and *Balaenella brachyrhynus* suggest that an expansion of the paleobiogeographic range of *Balaena*-like taxa was attained during the earliest part of the Pliocene with invasion of Mediterranean, North Atlantic and North Sea. The sister-group relationship of *Balaena mysticetus* and the other *Balaena-*like taxa suggest that the direct ancestor of the living bowhead whale originated around the Zanclean or slightly earlier and possibly quickly invaded the Arctic region, leaving us more limited possibilities to find fossil records relevant for the morphological transition toward the extant species.

The stratigraphic age of *Eubalaena shinshuensis* is the most crucial point in the present reconstruction of the divergence dates of balaenoid taxa. In fact, as the occurrence of this species is from the Messinian, the origin of the whole *Balaenula + Eubalaena* clade must be traced back to at least the latest Miocene. This means that the separation of the living right whales from their closest living relative (i.e., *Balaena mysticetus*) is from 7-to-5.4 million years ago which significantly increases the hypothesized divergence date based on McLeod, Whitmore & Barnes (1993) and reduces to one-third of the hypothesized divergence date based on Bisconti (2005b) and Santangelo et al. (2005). The impact of this new divergence date on the reconstruction of the demographic history of the right whales based on genetic measures of diversity will be analyzed elsewhere.

As far as the origins of the living *Eubalaena* species is concerned, the Messinian age of *Eubalaena shinshuensis* suggests that the origin of the genus *Eubalaena* should be found at least in the latest Miocene. The stratigraphic occurrences of the *Eubalaena* sp. from Tuscany and *Eubalaena ianitrix* constrain the origin of the living right whale species to at least the Piacenzian. Therefore, we estimate that the modern *Eubalaena* species originated in a period between 3.5 and 2.6 Ma. As we will show in another paragraph, it is more difficult to determine a chronological placement for the origin of the northern right whale *Eubalaena glacialis* because the origin of this species could be due to a process of phyletic transformation from *Eubalaena ianitrix*, occurring in a time interval ranging from the Piacenzian to the Pleistocene.

In summary, the stratigraphic distribution of the main evolutionary events of Balaenoidea are presented in Fig. 13. Following our phylogenetic analysis and the computation of stratigraphic ages of the included OTUs, the origin of Balaenoidea should be traced at least as far as the Burdigalian (age of *M. parvus*). The origin of the living families (Neobalaenidae and Balaenidae) occurred before the Tortonian (age of *Miocaperea pulchra*). The splitting between the *Balaena*-like and the *Balaenula + Eubalaena* clades occurred before the Messinian (age of *Eubalaena shinshuensis*). The origin of the modern *Eubalaena* radiation (including *Eubalaena ianitrix*) dates at least from the Piacenzian. The separation between the living right whale species and their extant relative (*Balaena*) dates at least from the earliest Messinian (*c.* 7 Ma).

## Discussion

### Body size estimate

The methods we used to estimate the size of *Eubalaena ianitrix* resulted in a total body length included between 6 and 7 m and a body mass of *c.* 20 t. The statistical methods used have their shortcomings in that most of them were not tested on species of the genus *Eubalaena*. The ratio between skull length and body length is a general estimate of body proportions in Balaenidae used by several authors based on observations of mounted skeletons, and killed and stranded animals (Koshi et al., 1993; Tomilin, 1967; Omura, 1958). The ratio between supraoccipital length and total skull length was used by Bisconti (2002) based on a small dataset of right whale measurements and its correlation coefficient *R*^2^ is rather low; therefore, the body size estimate generated by this method must be considered as preliminary pending the inclusion of more measurements in the dataset. However, all the methods used converge toward a total body length of 6-to-7 m and we think that this result should be close to the true length of the living animal. At present, we have no reason to suppose that a kind of systematic error occurred in a consistent manner to provide a systematically wrong result based on all the methods used.

It is unclear whether this size represents the maximum length of *Eubalaena ianitrix* because nothing is known about its individual variation. If compared with other balaenids, it represents a medium-sized species (see Table S2; Fig. S1). More precisely, it is the only medium-sized species within the *Eubalaena* clade (Fig. S2), suggesting that its medium size is a derived condition. The origin of the size reduction in *Eubalaena ianitrix* may be related to the warmer temperature of the southern portion of the North Sea during the deposition of the Lillo Formation (latest Zanclean-early Piacenzian; Laga, Louwye & Mostaert, 2006). In fact, the Kruisschans Sands Member of the Lillo Formation (in which the holotye skull of *Eubalaena ianitrix* was found) deposited in a shallow, low-energy environment, where molluscs indicate some degree of cooling, but where the palynological assemblage suggests mild-temperate to warm marine conditions (Marquet, 1993; Louwye, Head & De Schepper, 2004; De Schepper, Head & Louwye, 2009). However, it is still unknown whether *Eubalaena ianitrix* inhabited permanently the southern North Sea.

In the extant *Eubalaena glacialis* and *Eubalaena australis*, a total body length of less than 7 m corresponds to the length of individuals less than one year old (George et al., 2016; Fortune et al., 2012). The holotype skull of *Eubalaena ianitrix*, however, shows sutural morphologies and general robustness inconsistent with the general osteological features of newborn and early juvenile individuals (e.g., occipital joints not closed, presence of spongy bone; see Walsh & Berta, 2011). Rather, its robust muscular attachments on the supraoccipital and the degree of fusion at the frontoparietal (coronal) and parietal–squamosal sutures suggests that its age was older than one year. It is impossible to assess at which stage of its life cycle it died as nothing is known about intraspecific variation in skull and body length in *Eubalaena ianitrix*. Future discoveries of new specimens from different age classes will help providing an overview of the ontogenetic variation in body size in this newly discovered species.

The estimate of the body mass obtained in the present work is at odds with published records regarding the relationships of body mass and total body length in extant Balaenidae. Fortune et al. (2012) reported weights ranging from 0.7 to 11 t for 6–9 m long bowhead whales, while Trites & Pauly (1998) estimated masses ranging from 19 to 24 t for 16–18 m long northern and southern right whales. Our result of *c.* 20 for the 6–7 m long *Eubalaena ianitrix* appears overestimated, suggesting that more research is needed to develop more accurate statistical methods for inferring body size and body mass information in Balaenidae.

### Phylogeny: relationships of Balaenidae

Phylogenetic analyses of Balaenidae were published by several authors in the last 25 years. McLeod, Whitmore & Barnes (1993) were the first to publish a phylogenetic tree based on manual manipulation of morphological character states. They found a monophyletic Balaenoidea and a sister group relationship between Neobalaenidae and a clade formed by Balaenidae and Eschrichtiidae. The sister-group relationship of Balaenidae and Eschrichtiidae was not confirmed by subsequent phylogenetic works.

Bisconti (2000) performed the first computer-assisted cladistic analysis of Balaenidae, retrieving a monophyletic Balaenoidea and a monophyletic Balaenidae. Within Balaenidae, Bisconti (2000) found two different clades: one including the genus *Balaena* and the other including *Eubalaena*, *Balaenula* and *Morenocetus*. After a substantial re-discussion of the fossil record of Balaenidae (Bisconti, 2003) and of previously published phylogenetic analyses, Bisconti (2005a) published a new phylogenetic analysis resulting in a monophyletic Balaenoidea and a monophyletic Balaenidae; two clades were recovered in Balaenidae: one included *Morenocetus*, *Balaenella* and *Balaena* and the other included *Eubalaena* and *Balaenula*. These finds were substantially confirmed by Churchill, Berta & Deméré (2012) after an extensive reanalysis of the morphological evidence of the phylogeny of Balaenidae.

Numerous other works on the phylogeny of mysticetes were published in the last decades that included balaenids, but not explicitly focused on Balaenidae. However, it is important to consider these works as they provide information about the sister-group relationships of Balaenidae and other mysticete taxa. While most of the morphology-based works agree that Balaenidae and Neobalaenidae are sister-groups (Bisconti, 2015 and literature therein; Boessenecker & Fordyce, 2016), several recent papers did not support the monophyly of Balaenoidea with Neobalaenidae as sister group of Balaenopteroidea (see Gol’din, Startsev & Krakhmalnaya, 2014 and literature therein) or as part of Cetotheriidae (e.g., Marx & Fordyce, 2015 and literature therein). The placement of *Caperea marginata* and *Miocaperea pulchra* within Cetotheriidae depended upon peculiar treatments of some characters related to the shape and orientation of the squamosal, the elongation of the supraoccipital and the reduction of the ascending process of the maxilla. Criticisms to this approach were published by El Adli, Deméré & Boessenecker (2014) and Bisconti (2015) suggesting that the Marx & Fordyce (2015) dataset should be revised. Marx & Fordyce (2016) provided a subsequent version of such a dataset that shows substantially the same characteristics, as it does not include character states describing the topological relationships of the bones forming the skull vault in Balaenidae and Neobalaenidae. The proposed sister-group relationship of Neobalaenidae and Balaenopteroidea + Cetotheriidae depends on (1) inclusion of molecular data or (2) emphasis on rorqual-like characters observed in *Caperea marginata* (i.e., long forelimb, presence of dorsal fin, and presence of ventral throat grooves).

The problem of using molecular data to infer the phylogenetic relationships of clades mainly formed by fossil taxa (e.g., Hominidae, Mysticeti) has been addressed by several authors and this is the precise case of mysticetes where most of the described species are now extinct and cannot be used for DNA sequencing and analysis. Even if molecular analyses may include thousands of character states (base pairs from DNA sequences), the lack of data from most of the taxa belonging into the clade may be a serious problem as an enormous number of character states cannot be scored and must be inferred by the computer program used for the analysis. The accuracy of phylogenetic reconstructions based on molecular data for clades mainly formed by extinct taxa tends to be lower than that based on morphological data (Heath et al., 2008; Wagner, 2000). This suggests that emphasis should be given to morphological rather than molecular data in the inference of the phylogeny of mysticetes.

Aside from that, it must be said that taxonomic uncertainties, problems with character descriptions and coding, and the discovery of large amounts of homoplasy in morphological datasets have plagued morphological attempts to infer phylogenetic relationships in mysticetes in the last twenty years (Deméré, Berta & McGowen, 2005; Bisconti, 2007). Our effort to reduce dataset homoplasy was successful only in part. In fact, after the exclusion of evident homoplastic characters from our morphological dataset, the number of usable character states dropped down and our morphological evidence could provide only strong support for only some clades. Most of the species-level sister group relationships are thus supported by reduced numbers of synapomorphies. In this sense, what we observe in balaenid phylogenetics resembles what was observed in complicated analyses of evolutionary radiations occurring in relatively recent times (e.g., cichlid fishes and hominins; Seehausen, 2006; Haile-Selassie, Melillo & Su, 2016) with the important difference that the evolutionary radiation of Balaenidae occurred in a longer time interval. However, studies of DNA substitution rates interestingly showed that mysticete DNA evolves much more slowly than that of other mammals (Rooney, Honeycutt & Derr, 2001); therefore, only limited morphological change should be expected to occur in this group in the last few million years. This expectation is somewhat confirmed by the substantial stasis detected in the last 10 million years of neobalaenid evolution (Tsai & Fordyce, 2015; Bisconti, 2012) and by the small amount of morphological diversity observed in Balaenidae as discussed in this work. The reasons of the slow evolutionary pace in Balaenidae are not completely understood; one character that could be correlated is the evolution of increased individual longevity, demonstrated to be linked to DNA preservation (Jackson et al., 2009; Keane et al., 2015), which, in its turn, should reduce the accumulation of mutations preventing the evolution of phenotypic diversity.

### Phylogeny: intra-family relationships within Balaenidae

Published studies specifically directed at discovering phylogenetic relationships of Balaenidae recently converged toward the subdivision of this family into two sub-clades: a clade including *Balaena* and *Balaenella* and a clade including *Balaenula* and *Eubalaena*. These two groups are well supported by morphological characters and correspond to two different skull structures (as evidenced by Miller (1923), Kellogg (1928), McLeod, Whitmore & Barnes (1993), Bisconti (2005a)).

The contribution of the postcranial skeleton to the support for these clades is rather scanty but, for the first time, we detected that: (1) the dorsal transverse process of the atlas is dorsoventrally enlarged in *Eubalaena* and reduced in *Balaena* (including *Balaena mysticetus* and *Balaena ricei*), (2) the ventral transverse process of the atlas is long and forms a ventral corner in *Balaena* but is short and squared in *Eubalaena*, (3) the humerus is long and slender in *Balaena* while in *Eubalaena* it is shorter and with a more globular head and (4) the dorsal corner of the olecranon process of the ulna is conspicuous in *Balaena* but reduced to absent in *Eubalaena*.

The four characters outlined above could be useful to suggest phylogenetic and taxonomic affinities of fossils of uncertain position because of their poor preservation. This is the case of a number of partial skeletons from the Pliocene of Italy (Bisconti, 2003; Chicchi & Bisconti, 2014; Cioppi, 2014; Bisconti & Francou, 2014; Manganelli & Benocci, 2014; Sarti & Lanzetti, 2014) that should be reassessed based on this new evidence.

As mentioned above, the sister-group relationship of *Balaena montalionis* and *Balaenella brachyrhynus* raises particular problems as the inclusion of *Balaenella* within *Balaena* would either make the latter paraphyletic, or would imply the assignment of *Balaenella* to *Balaena*. However, we feel that it is premature to choose one of the above options, as some morphological data from *Balaena ricei* were not available for this study (i.e., precise sutural pattern between parietal and frontal and between parietal and squamosal and shape of the anterior end of the supraoccipital); this makes relationships within the *Balaena*-like subclade still biased by some uncertainty. However, the close relationship of *Balaena montalionis* and *Balaenella brachyrhynus* seems well supported by the shared squared anterior border of the supraoccipital and the transverse compression observed in the anterior half of the lateral borders of the supraoccipital. The point, here, consists in understanding if *Balaena ricei* is more closely related to *Balaena mysticetus* or to the *Balaena montalionis + Balaenella brachyrhynus* pair; more data are needed about the morphology of *Balaena ricei* to solve this question.

Among right whales, *Eubalaena shinshuensis* is the first to branch off, due to the primitive sutural pattern observed in the skull of this Messinian species; the following branch is occupied by the Piacenzian *Eubalaena* sp. from Tuscany, due to the plesiomorphic parietal–squamosal suture and to a peculiar supraoccipital morphology. More interesting are the relationships of the living *Eubalaena* species and *Eubalaena ianitrix*. From our work, *Eubalaena japonica* is the earliest-diverging species among the living right whales, with *Eubalaena australis* and *Eubalaena glacialis* more closely related to each other. This result contradicts molecular studies that suggested that *Eubalaena australis* diverged earlier and that *Eubalaena glacialis* and *Eubalaena japonica* are sister-groups (Gaines et al., 2005; Rosenbaum et al., 2000). Also, the DNA-based phylogeny of species of lices parasitizing living *Eubalaena* species lends support to the molecular hypothesis of relationships for right whales (Kaliszewska et al., 2005) thus suggesting that *Eubalaena glacialis* is the earliest-diverging *Eubalaena* species. However, these analyses did not include data from fossil right whales such as *Eubalaena shinshuensis*, *Eubalaena ianitrix*, and the *Eubalaena* sp. from Tuscany and did not take into account the fossil histories of the different lice species; therefore, they could be unable to retrieve correct results (in accordance with Heath et al., 2008; Wagner, 2000). Moreover, assuming an early branching of *Eubalaena glacialis* in the phylogeny of the living right whales implies that reticulate biogeographic histories have occurred between the southern and the North Pacific *Eubalaena* species to account for the peculiar genetic patterns observed in cyamid lices (Kaliszewska et al., 2005).

### Divergences of the living right whale species

Divergence ages of living balaenid species are important for the reconstructions of the demographic histories of these taxa in the context of conservation biology. Divergence dates are used in equations dealing with the genetic diversity of the living populations to assess whether living species suffered of genetic bottlenecks due to environmental change or human impact (Rosenbaum et al., 2000). Fossil calibrations of divergence dates are necessary to constrain the pace of molecular clocks in order to get correct results in terms of assessments of genetic diversity and evolution (Quental & Marshall, 2010).

Several works have provided estimates of divergence ages of balaenid species. McLeod, Whitmore & Barnes (1993) suggested a separation date between *Eubalaena* and *Balaena* of *c.* 4.5 Ma based on analysis of the balaenid fossil record. This assessment was used by Rosenbaum et al. (2000) to analyze the genetic diversity of the living bowhead whale, *Balaena mysticetus*, with the conclusion that this species did not suffer of population bottlenecks due to human whaling activities. Bisconti (2005b) and Santangelo et al. (2005) questioned this conclusion based on the phylogenetic analysis provided by Bisconti (2005a); the latter opened the possibility that the divergence between *Eubalaena* and *Balaena* occurred in the Early Miocene. This conclusion resulted from the placement of *M. parvus* as sister-group of the *Balaena-*like subclade to the exclusion of the *Balaenula + Eubalaena* subclade (Bisconti, 2005a) thus providing a divergence date of *Balaena* and *Eubalaena* of more than 20 Ma.

Subsequent analyses did not confirm this result as molecule-based and morphology-based works suggested later divergence dates (Sasaki et al., 2005; Churchill, Berta & Deméré, 2012) and placed the divergence of *Balaena* and *Eubalaena* in a time interval ranging from *c.* 4 to *c.* 7 Ma. The phylogenetic analysis of *Cyamus* lices confirms a divergence at *c.* 6.6 Ma for the living right whale and bowhead whale species (Kaliszewska et al., 2005).

The phylogenetic analysis of the present work (Figs. 12 and 14) reinforces a minimum late Miocene divergence (Messinian: *c.* 7–5.4 Ma) based on the age of the earliest diverging *Eubalaena* species (i.e., *Eubalaena shinshuensis*). In fact, an earlier divergence age is not unlikely, considering that (1) based on the present work, the separation between Balaenidae and Neobalaenidae dates from at least the Tortonian (*c.* 10 Ma) and (2) the separation of the *Balaena*-like subclade from the *Balaenula + Eubalaena* subclade is deep in time and originates from the very origin of Balaenidae (Bisconti, 2005a; Churchill, Berta & Deméré, 2012; this work).

How the reconstructions of the demographic histories of balaenids will be impacted by a Late Miocene age of divergence between *Eubalaena* and *Balaena* is outside the scope of the present paper. However, we suggest here that the past estimates of genetic diversity in right and bowhead whale populations should be considered with caution as those were based on underestimated (McLeod, Whitmore & Barnes, 1993) or overestimated (Bisconti, 2005b; Santangelo et al., 2005) divergence ages.

### Possible ancestor–descendant relationships between *Eubalaena ianitrix* and *Eubalaena glacialis*

There is not a commonly accepted method to infer ancestor–descendant relationships (ADRs) in phylogenetics as it is supposed that only in exceptional cases such a relationship can be detected in the fossil record (Paul, 1992). The most usual recommendation to those who try to recover ADR from the fossil record consists in being sure that a reasonably complete sample is available for the past diversity of the investigated group. While it is certain that this is not the case for fossil cetaceans, some attempts to reconstruct ADRs in this order were attempted in the past with a diversified array of methods.

Uhen & Gingerich (2001) provided an ADR for *Chrysocetus healyorum* and Neoceti (Mysticeti + Odontoceti). They used a stratocladistic approach in three steps: (1) they performed a traditional computer-assisted, morphology-based cladistics analysis retrieving a set of resulting cladograms; (2) they added a stratigraphic character and manipulated the initial hypothesis of relationships by hand in order to explore whether *Chrysocetus healyorum* could be the direct ancestor of Neoceti; (3) they calculated a new set of cladograms via a computer-assisted algorithm. They found one most parsimonious tree in which *Chrysocetus healyorum* was placed as direct ancestor of Neoceti. In the subsequent discussion, they suggested that newly discovered advanced archaeocete taxa could fit the ancestor position for Neoceti in a better way than *Chrysocetus healyorum* thus giving this taxon a temporary ancestor status.

More recently, Tsai & Fordyce (2015) suggested an ADR for *Miocaperea pulchra* and *Caperea marginata* based on a combination of cladistic analysis of traditional OTUs + juvenile individuals of *Caperea marginata* and by providing a discussion on the impact of the morphology of juvenile characters in phylogeny reconstruction. Apart from cetaceans, ADR were also hypothesized for the fur seal *Callorhinus* (Boessenecker, 2011), great white sharks (Ehret et al., 2012), and the dinosaur *Triceratops* (Scannella et al., 2014).

All of these methods have their own merits and shortcomings; Uhen & Gingerich (2001) realized a systematized search for the most parsimonious solutions but their results were limited by uncertainties about the completeness of the relevant fossil record; Tsai & Fordyce (2015) used data from a hotly debated source of data (i.e., juvenile and embryonic specimens) (e.g., Hall, 1996 and literature therein). Apart from that, however, the search for ADRs is always worth doing, as it potentially gives information on natural evolutionary processes.

Here, we suggest that an ADR should be proposed for the *Eubalaena ianitrix* and *Eubalaena glacialis* species pair. We support our hypothesis of relationships based on what follows:

1. *Eubalaena glacialis* and *Eubalaena ianitrix* are phylogenetically more closely related than all the other species belonging to *Eubalaena*; they share one peculiar synapomorphy that is not observed in any other *Eubalaena* species (i.e., presence of the pterygoid in the temporal fossa).
2. Molecular studies suggest that the branch of *Eubalaena glacialis* has been separated from the other living right whale species for a long time (up to three million years). This long time interval excludes the possibility of an arrival in the North Atlantic due to a Plesistocene or Holocene invasion from the North Pacific or the southern right whale species (Kaliszewska et al., 2005). Thus, it is highly likely that *Eubalaena glacialis* originated in that portion of the northern hemisphere that includes the North Atlantic and the North Sea.
3. *Eubalaena glacialis* and *Eubalaena ianitrix* share part of their geographic distribution. Even if only one specimen of *Eubalaena ianitrix* is known up to now, its geographic occurrence is included within the geographic range of *Eubalaena glacialis*.
4. The geographic area that encompasses the distribution of *Eubalaena ianitrix* and *Eubalaena glacialis* underwent extensive environmental change during the past 1.5 million years (Zachos et al., 2001), supporting the hypothesis that selective regimes could have been active there implying phenotypic evolution in previously established populations. In particular, the temperature decline observed in the whole northern hemisphere during the Pleistocene could have been the driver of organismal responses that can be described (in part, at least) by the Bergmann’s rule (i.e., increasing body size).
5. Assuming a species longevity of two million years (Fordyce & de Muizon, 2001; Steeman et al., 2009), and hypothesizing that *Eubalaena glacialis* became a well-defined species around the Pliocene–Pleistocene boundary, there may be a time interval in which *Eubalaena ianitrix* and the earliest individuals of *Eubalaena glacialis* co-occurred in the same area where the morphological transition happened. Unfortunately, the estimated species longevity mentioned above is only based on the observation that the fossil record of the living mysticete species does not exceed *c.* two million years. Based on molecular data, alternative analyses suggest longer species durations (see Pastene et al. (2007) for *Balaenoptera acutorostrata* and Sasaki et al. (2005) for many species of baleen-bearing whales; these studies suggest divergence dates of some living species exceeding two million years). This does not contradict our proposed sympatry hypothesis for *Eubalaena glacialis* and *Eubalaena ianitrix*; rather, hypothesizing longer species duration would reinforce this hypothesis. To our knowledge, no molecule-based work supports a species duration shorter than two million years for extant baleen-bearing whales.
6. Bearing in mind the paleoenvironmental changes that occurred in the northern hemisphere from the earliest Pleistocene (*c.* 2.6 Ma) through most of that epoch, a transformation of a previously established population of right whale into a more ecologically optimized species is a reasonable hypothesis.
7. From a skeletal morphology perspective, if a phyletic transformation of *Eubalaena ianitrix* into *Eubalaena glacialis* occurred, then it involved: (i) massive size increase at adulthood enabling the extant *Eubalaena glacialis* to reach more than 20 m in length at maturity (Tomilin, 1967) against the *c.* 7 m of *Eubalaena ianitrix* (consistent with Bergmann’s rule in a colder environment), (ii) possible allometric adjustments of bone proportions (this is a direct consequence of point 1), (iii) loss of the crest at the parietal–squamosal–supraoccipital suture and (iv) change in the orientation of the posteromedial corner of the palatine. The crest at parietal–squamosal–supraoccipital suture appears to have been lost in the common ancestor of *Eubalaena glacialis + Eubalaena ianitrix + Eubalaena japonica + Eubalaena australis* clade and its presence in *Eubalaena ianitrix* is to be interpreted as a reversal to a plesiomorphic condition. The same applies to the protrusion of the posteromedial corner of the palatine. The recurrent evolution of these two characters suggests that some homoplasy occurred in the above clade in the last few million years.
8. Current genetic evidence supports the view that three distinct species of right whales inhabit three different ocean basins (Malik et al., 2000; Rosenbaum et al., 2000): *Eubalaena glacialis* in the North Atlantic and adjacent waters, *Eubalaena japonica* in the North Pacific, and *Eubalaena australis* in the Southern Ocean. Balaenoid whales perform a particular feeding behavior directed at capturing calanoid copepods; this feeding behavior is known as continuous ram feeing (Sanderson & Wassersug, 1993) or skim feeding (Pivorunas, 1979). In the northern hemisphere, there is a geographic separation between the skim feeding species: the bowhead whale inhabits Arctic waters, while the right whales inhabit more temperate waters and the two right whale species of the northern hemisphere are separated by the Eurasia and thus do not compete for food or reproductive areas. In the southern hemisphere, the two skim feeding species are geographically separated as the southern right whale feeds around Antarctica while the pygmy right whale is restricted to more temperate waters; apparently, there is no competition between these species for food or reproductive areas. It appears, thus, that only one skim feeding species is “allowed” to live in a given ocean basin, and we may hypothesize that the pattern was not different in the past million years. For this reason, we may expect that only one or a few right whale species occupied a given geographic area in time intervals of *c.* two million years (mean duration of a marine mammal species; see above). This suggests that, paradoxically, the taxonomic sample of the right whale diversity in the Late Pliocene of the northern hemisphere is rather complete. This inference is also confirmed by the high value of the SCI obtained here, suggesting that most of the phylogenetic relationships presented here can be explained without the need for long ghost lineages. This inference fills the requests for a dense taxonomic sampling in the taxa under investigation and allows us to give further support to our hypothesis of ADR for *Eubalaena ianitrix* and *Eubalaena glacialis*. It must be said, however, that the current diversity of right and bowhead whales includes only large-sized species, whereas, in the Pliocene, large-sized and small-sized balaenid species are demonstrated to have been sympatric (Bisconti, 2003). Moreover, several studies have addressed the impact of shark predation on Pliocene right whales, suggesting some differences in the trophic webs of the Pliocene oceanic basins with respect to modern times. The ecological meanings of these differences are still not fully understood, potentially impacting our hypothesis regarding the taxonomic completeness of the balaenid fossil record.
9. In a way to test the ADR for *Eubalaena ianitrix* and *Eubalaena glacialis*, we followed the stratocladistic approach of Uhen & Gingerich (2001). The taxon x character matrix and the single most parsimonious tree were taken to MacClade (Maddison & Maddison, 2000). First, without the addition of a stratigraphic character, the ADR for *Eubalaena ianitrix* and *Eubalaena glacialis* was demonstrated to increase the tree length of two steps, as compared to the original tree length with a sister-group relationship. After addition of the stratigraphic character (see Supplementary Information) and without any other modification of the topology, the difference in tree length decreased from two steps to one step, meaning that the sister-group relationship was still the most parsimonious, but that stratigraphic data, namely the Piacenzian age of *Eubalaena ianitrix*, made the difference less significant. Swapping branches by hand, ADR for *Eubalaena ianitrix* and *Eubalaena glacialis* was found more parsimonious than a sister-group relationship only with (i) *Eubalaena shinshuensis* being more stemward than *Balaenula astensis*, and (ii) the three extant *Eubalaena* species forming a clade, with *Eubalaena ianitrix* as their last common ancestor. The need for such changes in topology may indicate that *Eubalaena ianitrix* is not the ancestor of *Eubalaena glacialis*. However, we think that such a pattern is strongly impacted by the scanty Pliocene balaenid fossil record in some areas (e.g., the North Pacific and the Southern Ocean). Pending the future discovery of fossil relatives of *Eubalaena australis* and *Eubalaena japonica*, stratocladistic analyses will most likely not be able to unambiguously discriminate ADR and sister-group relationships for *Eubalaena ianitrix* and *Eubalaena glacialis*.

## Conclusion

We re-described specimens previously referred to “*Balaena*” *belgica* and found what follows.

1. The cervical complex RBINS M. 881 (IG 8444) that was originally designated as type of “*Balaena*” *belgica* by Abel (1941) is poorly preserved and does not show diagnostic characters below the family level; therefore, we assign it to Balaenidae gen. et sp. indet.; this decision makes “*Balaena*” *belgica*, and its recombination nomina dubia.
2. The fragment of maxilla RBINS M. 880 lacks crucial diagnostic characters and cannot be assigned to any of the described balaenid genera and species; it is therefore assigned to Balaenidae gen. et sp. indet.
3. The morphology of the humerus RBINS M. 2280 is closer to that of *Eubalaena glacialis* as compared to *Balaena mysticetus* in the shape of the articular facet for the olecranon process of the ulna, in the overall shape of the deltoid tuberosity, and in the shape of the posterior border of the diaphysis. However, it differs from *Eubalaena glacialis* and other extant *Eubalaena* species in the elongation of the straight posterior border of the diaphysis; it is therefore assigned to *Eubalaena* sp. indet. This humerus corresponds to a large individual reaching a total body length over 16.5 m; it represents the first report of a gigantic right whale in the fossil record of the North Sea.
4. The neurocranium RBINS M. 879a-f represents the holotype of the new species *Eubalaena ianitrix.* This species is described and analyzed into a phylogenetic context. From a morphological viewpoint, *Eubalaena ianitrix* is very close to the northern right whale *Eubalaena glacialis* in having the same sutural pattern in the skull vault and in sharing the presence of the pterygoid in the temporal fossa. From a phylogenetic view, *Eubalaena ianitrix* is the sister-group of *Eubalaena glacialis*.
5. Our phylogenetic analysis also retrieved a monophyletic Balaenoidea, with *M. parvus* as the earliest stem balaenoid taxon, and with Neobalaenidae being the sister-group of Balaenidae. Two clades are observed within Balaenidae: one including *Balaena-*like taxa (genera *Balaena* and *Balaenella*) and the other including *Balaenula* and *Eubalaena*. The Messinian *Eubalaena shinshuensis* is the earliest diverging *Eubalaena* species; the *Eubalaena* sp. from Tuscany is the sister-group of a clade including all the living *Eubalaena* species and *Eubalaena ianitrix*.
6. The separation of *Eubalaena* from *Balaena* is estimated to have occurred around 7 Ma (minimum age). The origins of the living right whale species should be chronologically constrained to the Piacenzian (Late Pliocene: at least between 3.6 and 2.6 Ma). Judging from supporting synapomorphies, stratigraphic ranges and ecological requirements, it is suggested that *Eubalaena ianitrix* is the direct ancestor of *Eubalaena glacialis*, the latter is proposed to have evolved via phyletic transformation, through body size increase and allometric adjustments during the temperature decline of the latest Pliocene and Pleistocene.

## Supplemental Information

10.7717/peerj.3464/supp-1 Supplemental Information 1 Supplementary information.

## Institutional abbreviations

AMNH: American Museum of Natural History, New York, USA
IZIKO: IZIKO Natural History Museum, Cape Town, South Africa
MSNT: Museo di Storia Naturale e del Territorio, Università di Pisa, Calci, Italia
NBC: Naturalis Biodiversity Center, Leiden, the Netherlands
RBINS: Royal Belgian Institute of Natural Sciences, Brussels, Belgium

Additional abbreviations are provided in the Supplementary Information File.

## Additional Information and Declarations

### Data Availability

The following information was supplied regarding data availability: The raw data has been supplied as Supplemental Dataset Files.

### New Species Registration

The following information was supplied regarding the registration of a newly described species: Publication LSID: urn:lsid:zoobank.org:pub:C8D3FE95-303E-4EF4-86DD-1B453E124981. Eubalaena ianitrix LSID: urn:lsid:zoobank.org:act:F17C4DCA-FF1B-4EA4-9E6B-6C1EED448745.
