# Supplementary material for: Revision of “Balaena” belgica reveals a new right whale species, the possible ancestry of the northern right whale, Eubalaena glacialis, and the ages of divergence for the living right whale species
Source: PeerJ. 2017 Jun 27;5:e3464. doi: 10.7717/peerj.3464 (PMC5490463; doi:10.7717/peerj.3464)
Supplement: Supplemental Information 1 [file peerj-05-3464-s001.pdf]

Supplementary information to

Revision of *'Balaena' belgica* reveals a new right whale species, the possible ancestry of the northern right whale, *Eubalaena glacialis*, and the ages of the divergence for the living right whale species

Michelangelo Bisconti,[1] Olivier Lambert[2] and Mark Bosselaers[2,3]

[1]San Diego Natural History Museum, Balboa Park, 1788 El Prado, San Diego, CA 92101, USA
[2]Royal Belgian Institute of Natural Sciences, Rue Vautier 29, 1000 Brussels, Belgium
[3]Zeeland Royal Society of Sciences, Middelburg, The Netherlands

Corresponding Author
Michelangelo Bisconti
Email address: michelangelobisconti@gmail.com

INDEX

Institutional abbreviations	Pag. 2
List of character states used in phylogenetic analysis	Pag. 3
Taxon x character matrix used in phylogenetic analysis	Pag. 8
Table S1. Stratigraphic ages of the taxa used in phylogenetic analysis	Pag. 12
Calculations of body length in extant and fossil balaenoid species	Pag. 13
Table S2. Raw data, methods of inference and range of total body length for the balaenoid species cited in the text.	Pag. 14
Supplementary Figure S1. Range of body lengths at adulthood across Balaenoidea based on Table S2.	Pag. 15
Supplementary Figure S2. Character reconstruction at internal nodes of Balaenoidea based on Fitch's (1971) parsimony.	Pag. 16
Literature cited in the Supplementary Information	Pag. 17

INSTITUTIONAL ABBREVIATIONS

AMNH, American Museum of Natural History, New York, USA.
IZIKO, Iziko South Africa Museum of Natural History, Cape Town, South Africa.
MSNT, Museo di Storia Naturale e del Territorio dell'Università di Pisa, Calci, Italia.
MZUSN, Museo Zoologico dell'Università degli Studi di Napoli, Napoli, Italia.
NBC, Naturalis Biodiversity Center, Leiden, The Netherlands.
RBINS, Royal Belgian Institute of Natural Sciences, Brussels, Belgium.
USNM, National Museum of Natural History, Smithsonian Institution, Washington DC, USA.

LIST OF CHARACTER STATES USED IN PHYLOGENETIC ANALYSIS

Rostrum: Premaxilla, maxilla, nasal

1) Position of posterior end of premaxilla: (0) on the anterior border of supraorbital process of frontal; (1) located more posteriorly and covering part of the interorbital region of the frontal.
2) Transverse expansion of anterior portion of premaxilla: (0) present; (1) absent.
3) Mesorostral groove: (0) absent; (1) present.
4) Dental ontogeny: (0) polyophiodonty; (1) monophyodonty.
5) Baleen plates: (0) absent; (1) present.
6) Baleen plates: (0) short; (1) long.
7) Plate-like infraorbital process of the maxilla: (0) absent; (1) present.
8) Lateral process of maxilla: (0) absent; (1) present.
9) Lateral process of maxilla: (0) short (broad-nosed rostrum); (1) long (sharp-nosed rostrum).
10) Teeth: (0) present on maxilla and/or premaxilla and mandible; (1) absent from maxilla, premaxilla and mandible.
11) Rostrum shape in lateral view: (0) rostrum straight; (1) rostrum highly arched.
12) Transverse compression of maxilla: (0) present as superior surface facing laterally and dorsally; (1) absent as superior surface faces dorsally.
13) Ascending process of maxilla: (0) absent; (1) present.
14) Ascending process of the maxilla: (0) short and narrow; (1) long and narrow; (2) short and wide.
15) Ascending processes of maxillae meeting posteriorly to nasals along the longitudinal axis: (0) no; (1) yes.
16) Location of posterior end of maxilla: (0) as posterior as posterior end of premaxilla; (1) more posterior than posterior end of premaxilla; (2) more anterior than posterior end of premaxilla.
17) Location of posterior end of ascending process of maxilla: (0) anterior to supraorbital process of frontal; (1) posterior.
18) Lateral border of ascending process of maxilla and posterior border of maxilla: (0) forming a right angle; (1) forming an obtuse angle.
19) Lateral borders of maxilla: (0) thick; (1) thin.
20) Infraorbital foramen disintegrated into a series of foramina: (0) no; (1) yes.
21) Contact between maxilla and frontal: (0) sutured along whole posterior border of maxilla; (1) sutured only along ascending maxilla.
22) Antorbital notch: (0) absent; (1) present.
23) Antorbital groove: (0) absent; (1) present.
24) Position of nasofrontal suture: (0) at anterior border of infraorbital region of frontal; (1) located well within interorbital region of the frontal.
25) Nasals transversely compressed: (0) no; (1) yes.
26) Posterior end of nasals converging medially: (0) no; (1) yes.
27) Position of anterior border of nasal: (0) at anterior end of skull; (1) at mid-length of rostrum; (2) in posterior quarter; (3) in line with anterior border of supraorbital process of frontal; (4) posterior to anterior border of frontal.

Jugal

28) Jugal short and ventrally convex: (0) no; (1) yes.

Frontal

29) Transverse elongation of supraorbital process of the frontal: (0) short; (1) moderate; (2) long.
30) Supraorbital process of the frontal: (0) horizontal; (1) gently descending from the interorbital region of the frontal; (2) slightly depressed from interorbital region of the frontal; (3) abruptly depressed from interorbital region of frontal.

31) Supraorbital process of the frontal projecting anteriorly: (0) no; (1) yes.
32) Shape of supraorbital process of the frontal: (0) flat; (1) prismatic.
33) Elongation of medial emergence of supraorbital process of frontal: (0) as long as distal border; (1) longer than distal border.
34) Anterior border of supraorbital process of frontal projected posterolaterally: (0) no; (1) yes.
35) Position of orbitotemporal crest: (0) at posterodorsal edge of supraorbital process of frontal; (1) on dorsal surface of supraorbital process of frontal; (2) at anterior border of supraorbital process of frontal.
36) Orbitotemporal crest shape: (0) sharply edged; (1) rounded; (2) reduced to a line.
37) Exposure of interorbital region of frontal: (0) long; (1) short because parietal is superimposed onto it; (2) short because posteromedial elements of rostrum are superimposed onto it.

Parietal

38) Shape of temporal fossa: (0) long and wide; (1) anteroposterior length shorter than transverse width.
39) Temporal fossa partially covered by lateral edges of supraoccipital: (0) no; (1) yes.
40) Shape of coronal suture: (0) transverse with respect to longitudinal axis; (1) anteriorly convex; (2) anteriorly concave.
41) Coronal suture systematically interdigitated with posteromedial elements of the rostrum: (0) no; (1) yes.

Squamosal

42) Glenoid fossa of the squamosal wide and allowing broad movements of mandible: (0) no; (1) yes.
43) Zygomatic process of squamosal longitudinally twisted and arched dorsally: (0) no; (1) yes.
44) Zygomatic process of squamosal with parallel medial and lateral borders: (0) no; (1) yes.
45) Secondary squamosal fossa: (0) absent; (1) present.
46) Supramastoid crest: (0) absent; (1) present.
47) Squamosal dorsoventrally elongated: (0) no; (1) yes.
48) Squamosal bulge: (0) present; (1) absent.
49) Systematic presence of squamosal cleft: (0) no; (1) yes.
50) V-shaped squamosal cleft: (0) absent; (1) present.

Occipital

51. Supraoccipital extending onto parietal posteriorly: (0) no; (1) yes.
52. Location of anterior end of supraoccipital: (0) posterior to anterior end of zygomatic process of squamosal; (1) anterior to anterior end of zygomatic process of squamosal.
53. Location of anterior end of supraoccipital: (0) posterior to or at level of orbit; (1) anterior to orbit.
54. Superimposition of supraoccipital on parietal: (0) short; (1) moderate as most of parietal is exposed in dorsal view; (2) moderate as only a subtle stripe of parietal is exposed in dorsal view; (3) massive as the parietal is not exposed in dorsal view because it is completely hidden by the supraoccipital; (4) the supraoccipital generates an anterior appendix that subdivides the parietal into two halves; (5) the supraoccipital subdivides the parietal into two halves by itself or by the interparietal bone.
55. Superimposition of supraoccipital on interorbital region of frontal: (0) absent; (1) present.
56. Shape of anterior border of supraoccipital: (0) broadly round; (1) triangular and pointed; (2) broadly squared; (3) narrowly squared.
57. Neck of occipital condyle: (0) well developed; (1) reduced.
58. Sites for attachment of neck muscles on supraoccipital: (0) moderately developed; (1) strongly developed.

59. Position of posterolateral corner of exoccipital in ventral view: (0) close to postglenoid process of squamosal; (1) posterolateral corner protruded posteriorly and medially reaching a point more posterior than the postglenoid process of squamosal; (2) posterolateral corner protruded posteriorly and laterally reaching a point more posterior than the postglenoid process of the squamosal; (3) posterolateral corner not protruded and reaching a point more anterior than the postglenoid process of the squamosal.

Basicranium

60. Palatal sulci and nutrient foramina: (0) absent; (1) present.
61. Foramen pseudovale: (0) absent; (1) present.
62. Hypoglossal foramen: (0) present; (1) absent.
63. Palatal maxillary sulci open into a long, alveolar groove: (0) absent; (1) present.
64. Massive elongation of palatine posteriorly: (0) absent; (1) present..
65. Pterygoid located towards posterior end of skull: (0) no; (1) yes.
66. Presence of median keel on palate: (0) no; (1) yes.

Petrosal

67. Shape of anterior process of petrosal: (0) broadly squared; (1) narrowly squared; (2) broadly round; (3) triangular.
68. Shape of lateral process of anterior process of petrosal: (0) tubercle-like; (1) triangular.
69. Crista transversa: (0) recessed in internal acoustic meatus; (1) not recessed and separating foramen for facial nerve from internal acoustic meatus at least in a phase of the ontogenetic process.
70. Transverse elongation of pars cochlearis of petrosal: (0) absent; (1) present.
71. Longitudinal elongation of pars cochlearis of petrosal: (0) absent; (1) present.
72. Median promontorial groove: (0) present; (1) absent.
73. Tegmen tympani: (0) relieved; (1) highly relieved; (2) low.
74. Roof of stylomastoid fossa: (0) crest-like; (1) no roof at all; (2) long and thick; (3) long and low.
75. Exposure of posterior process of petroympanics on skull in lateral view: (0) absent; (1) present.
76. Shape of posterior process of petrotympanics: (0) prismatic; (1) flat.
77. Anterior and posterior processes of petrosal fused to tympanic bulla: (0) no; (1) yes.

Tympanic bulla

78. Elliptical foramen: (0) present; (1) absent.
79. Distinct ventromedial ridge developed on tympanic bulla: (0) no; (1) yes.
80. Inner posterior prominence of tympanic bulla does not project posteriorly (therefore, interprominential notch of tympanic bulla is absent): (0) no, it projects; (1) yes.
81. Dorsoventral height of tympanic cavity: (0) high; (1) low.
82. Tympanic bulla dorsoventrally compressed: (0) no; (1) yes.
83. Epitympanic hiatus enlarged because of reduction of conical process in the tympanic bulla: (0) no; (1) yes.
84. Anterolateral lobe: (0) absent (pear-shaped bulla); (1) long; (2) short.
85. Ventral margin of sigmoid process of tympanic bulla: (0) present; (1) absent.

Mandible

86. Sharp myloyoidal groove: (0) absent; (1) present.
87. Distance between coronoid process and condyle: (0) short; (1) long.
88. Height of coronoid process of mandible: (0) high; (1) very short-to-absent; (2) middle sized.
89. Presence of postcoronoid crest and fossa in mandible: (0) no; (1) yes.

90. Postcoronoid crest and fossa well developed in mandible: (0) no; (1) yes.
91. Satellite process in mandible: (0) absent; (1) present.
92. Exposure of articular surface of mandibular condyle: (0) posterodorsal; (1) posterior; (2) dorsal.
93. Angular process of mandible projected posteriorly: (0) no; (1) yes.
94. Height and shape of angular process: (0) high; (1) middle sized; (2) low and rounded; (2) low and squared.
95. Dorsal bowing of dentary: (0) absent; (1) present and limited to anterior portion; (2) present along whole length.
96. Gingival foramina: (0) absent; (1) present.
97. Anterior torsion of dentary: (0) absent; (1) moderate; (2) strong.
98. Mental symphysis: (0) fused; (1) not fused.
99. Groove for mental ligament: (0) absent; (1) present.

Postcranial

100. Shortened cervical vertebrae: (0) no; (1) yes.
101. Cervical vertebrae fused (bodies and neural processes): (0) no; (1) yes.
102. Sacral vertebrae disintegrated: (0) no; (1) yes.
103. One pair of ribs articulated with sternum: (0) no; (1) yes.
104. Medial tuberculum of humerus absent: (0) no; (1) yes.
105. Articulation between humerus and radius+ulna: (0) rotational; (1) non rotational.
106. Radius+ulna and carpals: (0) in tight articulation; (1) not in tight articulation.
107. Anterior border of scapula projection: (0) dorsal; (1) anterior.
108. Posterior border of scapula projection: (0) dorsal; (1) posterior.
109. Hyperphalangy: (0) absent; (1) present.
110. Trapezium: (0) present; (1) absent.
111. Hindlimbs: (0) well developed; (1) strongly reduced.
112. Sternum: (0) formed by several sternebra; (1) formed by a single element.

Balaenidae module

113. Supraoccipital highly elongated: (0) no; (1) yes.
114. Sagittal crest on supraoccipital: (0) yes; (1) no: it is flat; (2) not it shows a sagittal concavity.
115. Dome on supraoccipital: (0) absent; (1) present.
116. Transverse compression of supraoccipital: (0) present; (1) absent.
117. Squared anterior border of supraoccipital: (0) no; (1) yes.
118. Transverse diameter of anterior border of supraoccipital: (0) short; (1) intermediate; (2) large.
119. Reduced zygomatic process of squamosal: (0) no; (1) yes.
120. Zygomatic process of squamosal projection: (0) anterolateral; (1) lateral.
121. Pterygoid present in temporal fossa: (0) no; (1) yes.
122. Foramen ovale with incomplete infundibulum: (0) no; (1) yes.
123. Orientation of supraorbital process of frontal: (0) posterior; (1) transverse.
124. Shape of orbitotemporal crest: (0) very low-to-absent; (1) sharp.
125. Shape of frontal-parietal suture: (0) straight; (1) with distinctive anteroventral corner.
126. Orientation of dorsoventrally elongated squamosal: (0) anterior; (1) posterior; (2) vertical.
127. Shape of exoccipital in lateral view: (0) round; (1) squared.
128. Height of ventral border or exoccipital in lateral view: (0) at orbit level; (1) higher than orbit.
129. Rostral curvature: (0) rostrum uniformly curved; (1) posterior part of rostrum horizontal and anterior part curved.
130. Concavity on anterior border of nasal: (0) absent; (1) present.
131. Short nasals: (0) no; (1) yes.
132. Crest at parietal-squamosal-supraoccipital interface: (0) absent; (1) present.

133. Parietal spreads onto emergence of supraorbital process of frontal: (0) no; (1) yes.
134. Shape of parietal-squamosal suture: (0) straight; (1) anteriorly protruded.
135. Shape of posterior process of petrosal in ventral view: (0) triangular; (1) squared; (2) prismatic.
136. Lateral process of anterior process of petrosal triangular: (0) no; (1) yes.
137. Size of lateral process of anterior process of petrosal: (0) narrow; (1) wide.
138. Transversely elongated pars cochlearis: (0) no; (1) yes.
139. Crista transversa within internal acoustic meatus: (0) yes; (1) no.
140. Vascular groove in posterior part of pars cochlearis: (0) yes; (1) no.
141. Pyramidal process evident posteriorly to perilymphatic foramen of petrosal: (0) present; (1) absent.
142. Size of endolymphatic foramen: (0) small; (1) large.
143. Height of transverse process of atlas: (0) short; (1) long.
144. Neural process on atlas neural arch: (0) present; (1) absent.
145. Shape of neural arch: (0) triangular; (1) round.
146. Anterior and posterior borders of humerus: (0) straight and parallel; (1) highly concave.
147. Humeral head: (0) small-sized; (1) globular and large-sized.
148. Deltopectoral crest location on humerus: (0) close to head; (1) located more distally.
149. Posterior expansion of distal end of ulna: (0) absent; (1) present.
150. Superior corner of olecranon: (0) present; (1) absent.
151. Coracoid process in scapula: (0) well developed; (1) reduced-to-absent.
152. Orientation of tyrohyoidal process: (0) posterior; (1) transverse.
153. Dorsoventral compression of anterior end of dentary: (0) moderate; (1) strong.

TAXON X CHARACTER MATRIX USED IN PHYLOGENETIC ANALYSIS

Protocetus atavus
00000-00- 0000--0--0 0000000000 0000000000 0000000010 -000-00000
0000000?-? 000??00000 00000?0?0?? ???????000 0??????????? ---------
- ---------- ---------- -----------

Georgiacetus vogtlensis
00000-00- 0000--0--0 0000000100 0000000000 0000000000 -000-00000
000000??? 00???00000 0000000000- 0000000000 010???????0? ---------
- ---------- ---------- -----------

Dorudon atrox
00000-00- 0000--1--0 0000000100 0000000000 0000000000 -000-00000
0000000-0 0000?00000 0000000000- 0000000000 01000000010 ---------
- ---------- ---------- -----------

Mammalodon colliveri
0??10-110 0011001111 1101100200 0100000000 00?0000010 -100000000
011-0000-1 0000000000 0000000000- 000000000? 0??????????1 ---------
- ---------- ---------- -----------

Janjucetus hunderi
00010-110 0011000111 1101100210 0100000000 0000000010 -100001001
0??0000??? 00???00??? ????00000- 000000000? ???????????? ---------
- ---------- ---------- -----------

Aetiocetus weltoni
1011??110 0011100201 1101100210 0000100200 0011100000 -1000000?1
111000???? ???????000 0000000000- 00000011? ???????????? ---------
- ---------- ---------- -----------

Eomysticetus whitmorei
101?10??? ?010--2--1 ????1012?1 0000000200 0011110010 -100011111
?11??000-0 0010100001 0000000000- 0001010110 0???1?1????? ---------
- ---------- ---------- -----------

Yamatocetus canaliculatus
101110110 0011000101 100110?111 0000000000 0011110010 -100101101
11?0000??? ???????001 0000000000- 0001010110 01111?01???? ---------
- ---------- ---------- -----------

Tokarahia kauaeroa
0111101?? 0010--0--1 1???001101 0000000000 0011110010 -100201101
11?00001-0 0000100001 00000?0?0?? ?????????0 01??1?01???? ---------
- ---------- ---------- -----------

Caperea marginata
011111111 11110000-1 1101000212 2010111111 1010001111 0111310100
11110010-1 0000010110 1111210110- 0202211111 111111111111 1000101000
0000000000 0000-00000 00000000000

Miocaperea pulchra
011111111 11110000-1 1101000212 2010111111 1010001111 0111310100
111001??? 000??10??? ????????1?? ?????????? ???????????? 1100101000
0000000000 000??0???? ???????????

Morenocetus parvus
0??11??? ?1?12000-? ?1??0????2 1000111111 101???111? ?111310101
?????????? ?????????? ??????????? ?????????? ???????????? 0000102???
00?2?????? 0????????? ???????????

Balaenella brachyrhynus
011111111 11012000-1 1101000?2 1010111111 1010001110 -111313100
111?1?111? 000??01110 111121????? ?????????? ???????????? 0101020101
0001010001 001110???? ???????????
Balaena mysticetus
011111111 11010000-1 1101000212 1010111111 1010001110 -111310100
1111111010 0011200110 1111211110- 0202212111 111111001111 0101111111
0001010001 0011100001 01100010001
Balaena montalionis
0??111??? ?1012000-? 11??0002?2 1010111111 1010001110 -111313100
111?11???? ?????????? ??????????? ?????????? ???????????? 0101020101
0001010001 00???????? ???????????
Balaena ricei
011111111 11012000-1 11??000??2 1??????111 101??01110 -111310100
??1???10?0 0001?0?1?? ??????1???? ????212111 1??01100???1 0001111?01
0001010??1 00???00001 000000100?1
Eubalaena glacialis
011111111 11010000-1 1101000212 1010010111 1010001110 -110310100
1111111011 1001200110 1111211110- 0202212111 111111001111 0210112011
1012111110 1121111111 10011111110
Eubalaena australis
011111111 11012000-1 1101000212 1010010111 1010001110 -111310100
1111111010 1001200110 1111211110- 0202212111 111011001111 0210112001
1012111110 1021111110 10011111110
Eubalaena japonica
011111111 11012000-1 1101000212 1010010111 1010001110 -111310100
11111111?1 1001200110 1111211110- 0202212111 111011001111 0210112001
1012111110 1121110110 10011111100
Eubalaena shinshuensis
011111111 11012000-1 1101000?2 1010010111 10??01110 -111310100
111???1??? ?????????? ??????????? ?????????? ???????????? 011011????
100?011100 00???????? ???????????
Eubalaena sp. (Tuscany)
????????? ?????????? ?????????? ???????1?1 1010001110 -111310100
??1??????? ?????????? ??????????? ?????????? ???????????? 00101120??
??0211???1 ?0???????? ???????????
Eubalaena ianitrix
0???11??1 11?12000-? ?1??0????2 1010010111 1010001110 -111310100
?11?11???? ?????????? ??????????? ?????????? ???????????? 0210112011
101211???1 11???????? ???????????
Balaenula astensis
011111111 11010000-1 1101000?2 1010010110 1010001110 -110310100
110??110?? 00?0200110 1111211110- 020221211? ???????????? 0100102011
1100001100 001??000?? ??????????0
Eschrichtius robustus
111111110 1111100101 1110100311 2000011211 2111101001 1100402112
11100013-1 1012300110 1000110110- 1200111111 011111001111 ---------
- ---------- ---------- -----------

Eschrichtioides gastaldii
1?1111110 1011101111 11??1003?1 2000012210 2110001010 -100100112
1110001??? ?????0?110 1000110110- 1200111111 0??11??????? ---------
- ---------- ---------- -----------

Balaenoptera physalus
111110111 1011100101 1101100412 3001122211 2111101011 1111502100
1110001300 1112301110 10002102111 0103011111 011111111111 ---------
- ---------- ---------- -----------

Megaptera novaeangliae
111110111 1011100101 1101100412 3001022211 2111101011 1111500103
1110001300 1110301110 10001102111 0103011111 011111111111 ---------
- ---------- ---------- -----------

Archaebalaenoptera castriarquati
001110111 1011101101 1101100312 3001022210 211110101? ?110500112
111??????? ??????00??? ?????1021?? 010301111? ???????????? ---------
- ---------- ---------- -----------

Titanocetus sammarinensis
001110110 1011101111 1101100212 1110011110 1011101000 -100200103
1110001??? ?????0???? ?????102111 0113011111 0??????????? ---------
- ---------- ---------- -----------

Cophocetus oregonensis
101110110 1011200111 1111100212 1110011201 1011101000 -110211101
1110001 2?1 0011300110 20000102110 011101??10 11?11101111? ---------
- ---------- ---------- -----------

Aglaocetus moreni
101110102 0111201111 1011100212 1010011210 1011101000 -110201102
1110001??? ??????00??? ?????102110 011101111? ???????????? ---------
- ---------- ---------- -----------

Parietobalaena palmeri
101110110 1011200111 1101100212 1010011210 1011111010 -110201101
1110001201 0001300110 10000102110 0111011111 01?????????? ---------
- ---------- ---------- -----------

Diorocetus hiatus
101110112 1011200111 1101101212 1110011210 1011101010 -110201111
1110001200 0000300110 20000102110 0121011111 01??1111???? ---------
- ---------- ---------- -----------

Isanacetus laticephalus
111110112 1011200111 1101110212 1010011210 1011101010 -110201101
1110001200 0001300110 10001?0?1?? ?????1???1 01?????????? ---------
- ---------- ---------- -----------

Pelocetus calvertensis
101110112 1011200111 1101110212 1110011210 1011101000 -110201101
1110001200 0001300110 20001102110 0111011111 01?11111?1?1 ---------
- ---------- ---------- -----------

Uranocetus gramensis
101110111 1011011101 1101111212 1010011210 2111101010 -110201100
1110001??0 0001?00110 10001102 1?? 0111011?1 0??11?00???1 ---------
- ---------- ---------- -----------

‘Aglaocetus’ patulus
001110112 1011000011 1101001212 1010011210 1011101000 -111201100
1110001200 0001300110 10001?0???? ?????????1 01?????????? ---------
- ---------- ---------- -----------

Piscobalaena nana
101110110 1011111111 1110111411 1110000210 2111101000 -100200102
1110001201 0000310110 10000102110 0021011111 011111111111 ---------
- ---------- ---------- -----------

Herpetocetus morrowi
1?1110110 0111111111 1111111211 1010001210 1011101000 -100201102
1110001311 0000?10110 10000102110 0021011111 01?????????? ---------
- ---------- ---------- -----------

Herenthalia nigra
1???????? ???111111? 11??111??? 1??0?11210 21?????001 01?0200102
?11?00???? 00????0??? ??????0???? ?????????? ???????????? ---------
- ---------- ---------- -----------

Tranatocetus argillarius
0???????? ???111110? ?1??110??? ???????210 2111101001 0100200101
?11000?30? 00???10110 10000?02??? 0????1???1 01?11?11???? ---------
- ---------- ---------- -----------

Mixocetus elysius
101110110 1011100111 1110101311 1110011210 2111101000 -100200101
1110001??? 00???????? ??????02??? 0????1???? ???????????? ---------
- ---------- ---------- -----------

Table S1.

Stratigraphic ages of the taxa used in phylogenetic analysis

taxon	Geological age	age (Ma)	Stratigraphic character state	Reference
Protocetus atavus	Lutetian (Early Eocene)	48.6-40.4	0	Paleobiology Database
Dorudon atrox	Priabonian (Late Eocene)	37.2-33.9	1	Paleobiology Database
Georgiacetus vogtlensis	Lutetian (Middle Eocene)	40.4-37.2	0	Paleobiology Database
Mammalodon colliveri	Chattian (Late Oligocene)	28.4-23.03	2	Paleobiology Database
Janjucetus hunderi	Chattian (Late Oligocene)	28.4-23.03	2	Paleobiology Database
Aetiocetus weltoni	Chattian (Late Oligocene)	28.4-23.03	2	Paleobiology Database
Eomysticetus whitmorei	Chattian (Late Oligocene)	28.4-23.03	2	Paleobiology Database
Tokarahia kauaeroa	Chattian (Late Oligocene)	27-25	2	Boessenecker & Fordyce 2015
Yamatocetus canaliculatus	Chattian (Late Oligocene)	28.4-23.03	2	Paleobiology Database
Morenocetus parvus	Burdigalian (Lower Miocene)	20.43-15.97	3	Paleobiology Database
Caperea marginata	Recent (Holocene)	0.012-0.0	8	Paleobiology Database
Miocaperea pulchra	Tortonian (Lower Miocene)	11.608-7.246	5	Bisconti (2012)
Balaena mysticetus	Recent (Holocene)	0.012-0.0	8	Paleobiology Database
Balaena ricei	Zanclean (Early Pliocene)	5.332-3.6	6	Paleobiology Database
Balaena montalionis	Zanclean (Early Pliocene)	5.332-4.0	6	Bisconti (2000)
Balaenella brachyrhynus	Zanclean (Early Pliocene)	5.332-3.6	6	Bisconti (2005)
Balaenula astensis	Piacenzian (Late Pliocene)	3.8-3.4	7	Bisconti (2000)
Eubalaena shinshuensis	Messinian (Upper Miocene)	7.246-5.332	5	Paleobiology Database
Eubalaena sp. (Tuscany)	Piacenzian (Late Pliocene)	3.8-3.6	7	Bisconti (2002)
Eubalaena japonica	Recent (Holocene)	0.012-0.0	8	Paleobiology Database
Eubalaena australis	Recent (Holocene)	0.012-0.0	8	Paleobiology Database
Eubalaena ianitrix	Piacenzian (Upper Pliocene)	3.2-2.8	7	This work
Eubalaena glacialis	Recent (Holocene)	0.012-0.0	8	Paleobiology Database
Eschrichtioides gastaldii	Zanclean-Piacenzian	5.332-2.588	6	Bisconti (2008)
Eschrichtius robustus	Recent (Pleistocene-Holocene)	2.5-0.0	8	Paleobiology Database
Archaebalaenoptera castriarquati	Piacenzian (Late Pliocene)	3.8-2.588	7	Paleobiology Database
Megaptera novaeangliae	Recent (Pleistocene-Holocene)	0.781-0.0	8	Paleobiology Database
Balaenoptera physalus	Recent (Holocene)	0.012-0.0	8	Paleobiology Database
Titanocetus sammarinensis	Langhian (Middle Miocene)	15.97-13.65	4	Paleobiology Database
Mixocetus elysius	Tortonian (Upper Miocene)	11.608-7.246	5	Paleobiology Database
Tranatocetus argillarius	Tortonian (Upper Miocene)	11.608-7.246	5	Paleobiology Database
Herentalia nigra	Tortonian (Upper Miocene)	11.608-7.246	5	Bisconti (2015)
Herpetocetus morrowi	Piacenzian (Late Pliocene)	3.8-2.588	7	Paleobiology Database
Piscobalaena nana	Messinian (Late Miocene)	7.246-5.332	5	Paleobiology Database
Cophocetus oregonensis	Burdigalian (Lower Miocene)	20.43-15.97	3	Paleobiology Database
Aglaocetus moreni	Burdigalian (Lower Miocene)	20.43-15.97	3	Paleobiology Database
Parietobalaena palmeri	Langhian (Middle Miocene)	15.97-13.65	4	Paleobiology Database
Isanacetus laticephalus	Burdigalian (Lower Miocene)	20.43-15.97	3	Paleobiology Database
'Aglaocetus' patulus	Langhian (Middle Miocene)	15.97-13.65	4	Paleobiology Database
Uranocetus gramensis	Tortonian (Upper Miocene)	11.608-7.246	5	Paleobiology Database
Pelocetus calvertensis	Langhian (Middle Miocene)	15.97-13.65	4	Paleobiology Database

CALCULATION OF BODY LENGTH IN EXTANT AND FOSSIL BALAENOID SPECIES

Data on body length of extant Balaenidae and Neobalaenidae species are reported in literature (Omura, 1958; Tomilin, 1967). Data on skull length and body length of extinct Balaenidae and Neobalaenidae were inferred based on the known skull/body proportions (from 1:3 to1:4) and on the relationship between supraoccipital length and skull length (Bisconti, 2002).
The body length of *Morenocetus parvus* was inferred based on a supraoccipital length of 1.6 m as provided by Buono (2013). The total body length was inferred from this value and ranged from 4.8 and 6.2 m.
The body length of *Miocaperea pulchra* was reconstructed based on skull/body proportions that in Neobalaenidae are approximately 1:4. The skull length of *M. pulchra* is *c.* 1.1 m (Bisconti, 2012) thus the total body length is *c.* 4.4 m.
The body length of *Balaena ricei* was estimated based on the supraoccipital length/skull length relationship. Being the supraoccipital length 0.67 m (Westgate & Whitmore, 2002), the total skull length is *c.* 1.6 m; the inferred total body length ranges from *c.* 4.8 and *c.* 6.4 m.
The body length of *Balaenella brachyrhynus* is inferred ranging from *c.* 3.3 to 4.4 m based on the total skull length (Bisconti, 2005).
The body length of *Balaena montalionis* ranged from *c.* 7.5 to *c.* 10 m based on a total skull length of *c.* 2.5 m (Bisconti, 2000).
The body length of *Balaenula astensis* ranged from *c.* 4.8 and *c.* 6.4 m based on an inferred skull length of 1.6 m (Bisconti, 2000).
The body length of *Eubalaena shinshuensis* ranged from *c.* 9.6 and *c.* 12.8 m based on a measured skull length of 3.2 m (Kimura, 2009).
The body length of the *Eubalaena* sp. from Tuscany ranged from *c.* 12.6 to *c.* 16.8 m based on an inferred skull length of 4.2 m (Bisconti, 2002).

Table S2

Raw data, methods of inference and range of total body length at adulthood for the balaenoid species cited in the text.

Taxon	Raw data[1] and references	Equation used	Body length range
Morenocetus parvus	SocL = 0.32 m (Buono, 2013)	Eq. (4) provides a skull length of 1.60 m[2]	*c.* 4.8-6.2 m
Miocaperea pulchra	SkL = 1.1 m (Bisconti, 2012)	Skull/body ratio[2]	*c.* 4.4 m
Caperea marginata	Data from literature (Baker, 1985)		*c.* 6-7 m
Balaenella brachyrhynus	SkL = 1.1 m (Bisconti, 2005)	Skull/body ratio[2]	*c.* 3.3-4.4 m
Balaena mysticetus	Data from literature (Tomilin, 1967; Reeves & Leatherwood, 1985)		16-22 m
Balaena ricei	SocL = 0.67 m (Westgate & Whitmore, 2002)	Eq. (4) provides a skull length of *c.* 1.6 m[2]	*c.* 4.8-6.4 m
Balaena montalionis	Data from literature (Bisconti, 2000)		*c.* 7.5-10.0 m
Balaenula astensis	Data from literature (Bisconti, 2000)		*c.* 4.8-6.4 m
Eubalaena shinshuensis	SkL = 3.2 m (Kimura, 2009)	Skull/body ratio[2]	*c.* 9.6-12.8 m
Eubalaena sp. (Tuscany)	Data from literature (Bisconti, 2002)		*c.* 12.6-16.8 m
Eubalaena japonica	Data from literature (Omura, 1958; Cummings, 1985)		*c.*15-18 m
Eubalaena australis	Data from literature (Omura, 1958)		*c.* 15-18 m
Eubalaena ianitrix	Present work		*c.* 5-7 m
Eubalaena glacialis	Data from literature (Omura, 1958; Cummings, 1985)		*c.* 16-18 m

[1]Explanation of acronyms: SkL, skull length (condylobasal length); SocL, supraoccipital length (from dorsal edge of foramen magnum to anterior end of supraoccipital). Data in meters.
[2]This work.

Supplementary Figure S1

Range of body lengths at adulthood across Balaenoidea based on Table S2.

Note that definitions of small, medium and large size are as follows: the size is small if total body length < 5 m; the size is medium if total body length is between 5 and 10 m; the size is large if total body length is > 10 m.

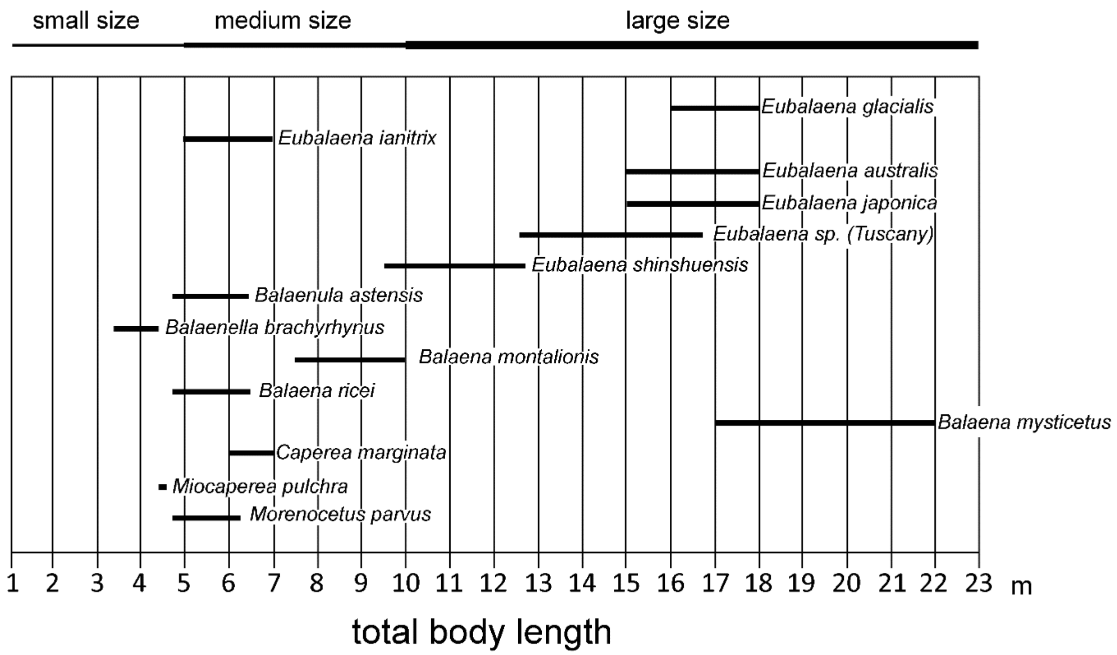

Supplementary Figure S2

Character reconstruction at internal nodes of Balaenoidea based on Fitch's (1971) parsimony.
As calculated, ancestral body size for Balaenoidea is medium; the evolution of large size is observed independently in *Balaena mysticetus* and the *Eubalaena* species with the exception of *E. ianitrix*. Small size is a derived character in *Balaenella brachyrhynus*, but it is unclear whether it is a derived character also in *Miocaperea pulchra*. On the right side, silhouettes are shown to visually illustrate the differences in the reconstructed body sizes among Balaenidae.

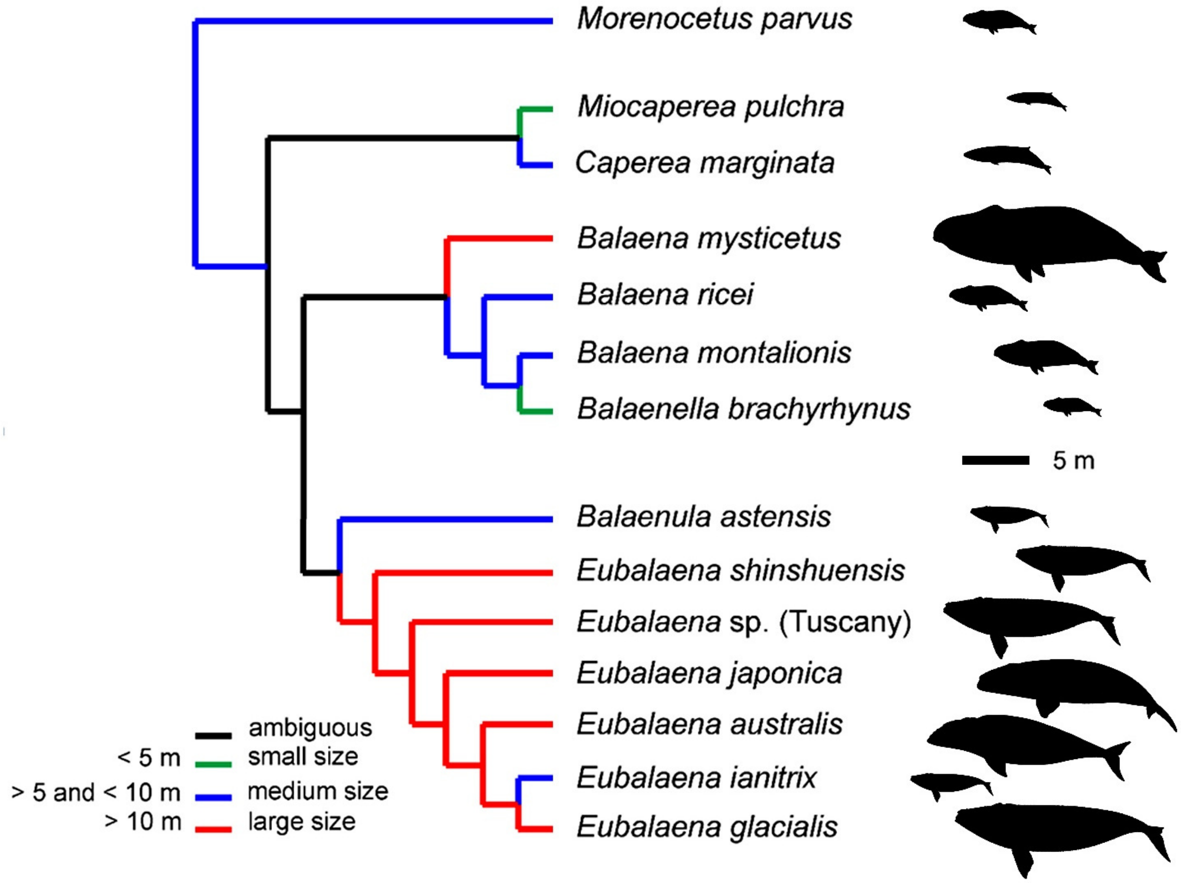

LITERATURE CITED IN THE SUPPLEMENTARY INFORMATION

Baker AN. 1985. Pygmy right whale *Caperea marginata* (Gray, 1846). In: Ridgway SH & Harrison R eds. *Handbook of marine mammals*. Vol. 3. London: Academic Press, 345–353

Bisconti M. 2000. New description, character analysis and preliminary phyletic assessment of two Balaenidae skulls from the Italian Pliocene. *Palaeontographia Italica* 87:37–66.

Bisconti M. 2002. An early late Pliocene right whale (Genus *Eubalaena*) from Tuscany (Central Italy). *Bollettino della Società Paleontologica Italiana* 4:83–91.

Bisconti M. 2005. Morphology and phylogenetic relationships of a new diminutive balaenid from the lower Pliocene of Belgium. *Palaeontology* 48:793–816.

Bisconti M. 2008. Morphology and phylogenetic relationships of a new eschrichtiid genus (Cetacea: Mysticeti) from the Early Pliocene of northern Italy. *Zoological Journal of the Linnean Society* 153:161–186.

Bisconti M. 2012. Comparative osteology and phylogenetic relationships of *Miocaperea pulchra*, the first fossil pygmy right whale genus and species (Cetacea, Mysticeti, Neobalaenidae). *Zoological Journal of the Linnean Society* 166:876–911.

Bisconti M. 2015. Anatomy of a new cetotheriid genus and species from the Miocene of Herentals, Belgium, and the phylogenetic and paleobiogeographic relationships of Cetotheriidae s.s. (Mammalia, Cetacea, Mysticeti). *Journal of Systematic Palaeontology* 13:377–395.

Boessenecker RW, Fordyce RE. 2015. A new genus and species of eomysticetid (Cetacea: Mysticeti) and a reinterpretation of *'Mauicetus' lophocephalus* Marples, 1956: transitional baleen whales from the Upper Oligocene of New Zealand. *Zoological Journal of the Linnean Society*. DOI: 10.1111/zoj.12297.

Buono MR. 2013. Evolucion de los Balaenidae (Mammalia, Cetacea, Mysticeti) del Mioceno de Patagonia: sistemática, filogenia y aspectos paleobiológicos. Dissertation Thesis. Universidad Nacional de La Plata.

Cummings WC. 1985. Right whales *Eubalaena glacialis* (Muller, 1776) and *Eubalaena australis* (Desmoulins, 1822). In: Ridgway SH & Harrison R eds. *Handbook of marine mammals*. Vol. 3. London: Academic Press, 275–304.

Kimura T. 2009. Review of fossil balaenids from Japan with a re-description of *Eubalaena shinshuensis* (Mammalia, Cetacea, Mysticeti). *Quaderni del Museo di Storia Naturale di Livorno* 22:3–21.

Omura H. 1958. North Pacific right whale. *The Scientific Reports of the Whale Research Institute Tokyo* 13:1– 52.

Reeves RR, Leatherwood S. 1985. Bowhead whale *Balaena mysticetus* Linnaeus, 1758. In: Ridgway SH & Harrison R eds. *Handbook of marine mammals*. Vol. 3. London: Academic Press, 305–340.

Westgate JW & Whitmore FC Jr. 2002. *Balaena ricei*, a new species of bowhead whale from the Yorktown Formation (Pliocene) of Hampton, Virginia. *Smithsonian Contributions to Paleobiology* 93: 295–312.
